# Source Apportionment of Aerosol at a Coastal Site and Relationships with Precipitation Chemistry: A Case Study over the Southeast United States

**DOI:** 10.3390/atmos11111212

**Published:** 2020-11-10

**Authors:** Andrea F. Corral, Hossein Dadashazar, Connor Stahl, Eva-Lou Edwards, Paquita Zuidema, Armin Sorooshian

**Affiliations:** 1 Department of Chemical and Environmental Engineering, The University of Arizona, Tucson, AZ 85721, USA; 2 Rosenstiel School of Marine and Atmospheric Science, University of Miami, Miami, FL 33149, USA; 3 Department of Hydrology and Atmospheric Sciences, The University of Arizona, Tucson, AZ 85721, USA

**Keywords:** positive matrix factorization, NADP, IMPROVE, CWT, source apportionment

## Abstract

This study focuses on the long-term aerosol and precipitation chemistry measurements from colocated monitoring sites in Southern Florida between 2013 and 2018. A positive matrix factorization (PMF) model identified six potential emission sources impacting the study area. The PMF model solution yielded the following source concentration profiles: (i) combustion; (ii) fresh sea salt; (iii) aged sea salt; (iv) secondary sulfate; (v) shipping emissions; and (vi) dust. Based on these results, concentration-weighted trajectory maps were developed to identify sources contributing to the PMF factors. Monthly mean precipitation pH values ranged from 4.98 to 5.58, being positively related to crustal species and negatively related to SO_4_^2−^. Sea salt dominated wet deposition volume-weighted concentrations year-round without much variability in its mass fraction in contrast to stronger seasonal changes in PM_2.5_ composition where fresh sea salt was far less influential. The highest mean annual deposition fluxes were attributed to Cl^−^, NO_3_^−^, SO_4_^2−^, and Na^+^ between April and October. Nitrate is strongly correlated with dust constituents (unlike sea salt) in precipitation samples, indicative of efficient partitioning to dust. Interrelationships between precipitation chemistry and aerosol species based on long-term surface data provide insight into aerosol–cloud–precipitation interactions.

## 1. Introduction

Aerosol–cloud interactions (ACI) are complex and not accurately represented by climate models [1]. These interactions remain the largest source of uncertainty in anthropogenic radiative forcing [2,3]. In contrast to subtropical regions characterized by stratocumulus decks such as off the western coasts of the United States (U.S.) [4], Chile [5], and Southern Africa (e.g., [6,7]), marine-related ACI studies are more scarce over the Western North Atlantic Ocean (WNAO) region [8].

Although few studies focusing on ACI over the WNAO have primarily relied on ship and/or aircraft data [8]. Even though these efforts have, in some cases, provided data at high temporal and spatial resolution and in the vicinity of clouds, downsides are the high cost, challenging logistics, and statistical limitations innate to short-term intensive campaigns. An alternative and indirect method to gain inferences about ACI is to use long-term data at surface monitoring networks gathering compositional data for precipitation and particulate matter. This method has been demonstrated for other regions such as an inland California site [9], the Southwestern U.S. [10], and Mexico City [11]. Those works demonstrated that colocated measurements of both precipitation and aerosol composition provide insight into some combination of the following processes: (i) the composition of particles serving as either the cloud condensation nuclei (CCN) or ice nuclei (IN) that eventually fall to the surface via wet deposition; (ii) uptake of species into existing droplets in cloud; and (iii) subcloud scavenging of different constituents. These three processes represent both aerosol effects on cloud (i) and cloud effects on aerosol (ii–iii). There are limitations when relating surface aerosol properties to precipitation at the same site, which are circumvented by airborne platforms flying around clouds; however, the ground-based approach leverages larger statistical datasets with broader temporal coverage that typically provide more speciation data.

For studies relating aerosol and precipitation chemistry, it helps to study locations with broad ranges in both aerosol and meteorological conditions. In this regard, coastal areas influenced by both natural and anthropogenic emissions are fitting as they are exposed to a wide assortment of aerosol types. On the western edge of the WNAO, Southern Florida fits into this category as it experiences influence from local and regional sources that are both biogenic and anthropogenic in nature [12], in addition to being under the impact of marine and shipping emissions [13], and long-range transport of dust [14] and smoke [15,16]. The area also experiences a wide range of precipitation, including from actively developing shallow cumulus clouds, which are common over the trade regions of most of the tropical oceans [17,18]. These clouds are especially important to study due to their radiative effect on climate model sensitivity [17]. Thunderstorms are prevalent during the summertime over the Gulf Coast and Florida, with the latter registering the highest concentration of thunderstorms in the U.S. [19]. Convergence from coastal sea breezes has been proposed as the dominant dynamic mechanism leading to thunderstorms during the summer months [20]. The combination of warm, moist maritime-tropical air and small-scale wind circulation (i.e., sea breeze) from both the Atlantic Ocean and the Gulf of Mexico provides the optimal conditions to form thunderstorms in this area.

Additionally, smaller bodies of water (e.g., Lake Okeechobee) can create circulations similar to sea breeze leading to conditions to form thunderstorms. Intercomparison of aerosol and wet deposition chemical data for Southern Florida can suggest what aerosol types participate in CCN/IN activation and what the resultant composition is of the wet deposition derived from clouds. Wet deposition flux magnitudes of different species are relevant for understanding impacts on surface ecosystems, which was already an active research area decades ago in the study region as part of the Florida Atmospheric Mercury Study [21,22].

This work aims to report on long-term aerosol and precipitation chemistry measurements from colocated monitoring sites at the Everglades National Park (NP) in Southern Florida. The analysis combined field measurement data, transport modeling, and source apportionment modeling to characterize the temporal trends and interrelationships between aerosol and precipitation chemistry from 2013 to 2018. The results and discussion are structured as follows: (i) summary of meteorological profile; (ii) sources of PM_2.5_; (iii) precipitation chemistry profile; and (iv) interrelationships between precipitation and aerosol chemistry.

## 2. Methods

### 2.1. Site Description

**Figure 1.**
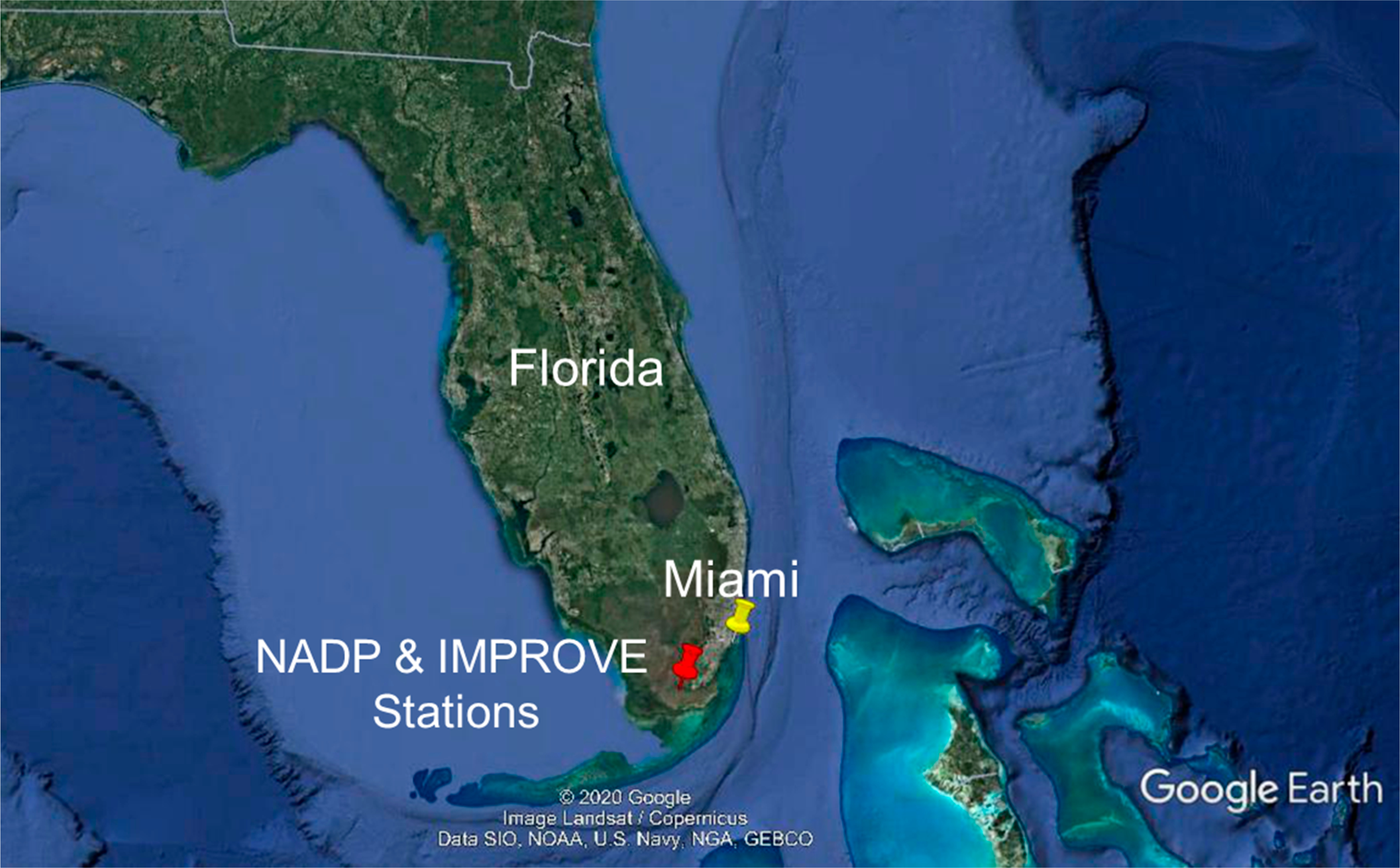
Map showing the location of Everglades National Park Interagency Monitoring of Protected Visual Environments (IMPROVE) and National Atmospheric Deposition Program (NADP) stations (red marker) in relation to Miami (yellow marker). Image source: Google Earth.

The Everglades NP hosts the precipitation monitoring station (25.390° N 80.680° W, 2 m above sea level (m a.s.l.)) managed by the National Atmospheric Deposition Program/National Trends Network (NADP/NTN) and the Environmental Protection Agency (EPA) Interagency Monitoring of Protected Visual Environments (IMPROVE) aerosol monitoring station (25.391° N 80.6806° W, 1 m a.s.l.). Both sites are located approximately 25 km from the coast. The Everglades NP is a unique ecosystem formed by subtropical wetlands (Figure 1). Located approximately 90 km southwest of Miami (Miami-Dade County population = 2,761,581 for 2018) [23], Everglades NP extends 1.5 million acres across Southern Florida. Approximately 50% of the original area has been converted to agricultural land and urban development [24,25]. Currently, 397,000 acres around the Everglades NP and the southern tip of Lake Okeechobee area are used for commercial sugarcane crops, Florida’s most profitable crop [26]. A byproduct of sugarcane production is 7 million tons of dry sugarcane leaves that are removed and burnt before harvesting between October and late March [27]. This is a common practice in other sugarcane producing areas like Louisiana, U.S. [28], Cuba [29], and Brazil [30]. These activities consequently result in substantial biomass burning emissions [27,31].

### 2.2. Precipitation and Aerosol Composition

Established in 1977 by the U.S. State Agricultural Experiment Stations (SAES), the NADP/NTN (hereafter referred to as NADP) began monitoring wet deposition across the U.S. to measure its effects on the environment. The network continued to expand throughout the years and currently has over 260 sites with the Wisconsin State Laboratory of Hygiene at the University of Wisconsin-Madison as the NADP Program Office. In 1987, the IMPROVE program was set up with 30 monitoring sites located in national parks as part of the visibility monitoring network [32], which currently includes over 100 sites. This study’s data were retrieved from the NADP and IMPROVE stations located at the Everglades NP between January 2013 and December 2018.

The NADP site collected weekly precipitation samples for pH, conductance, and water-soluble ions concentrations (ammonium (NH_4_^+^), calcium (Ca^2+^), chloride (Cl^−^), magnesium (Mg^2+^), nitrate (NO_3_^−^), potassium (K^+^), sodium (Na^+^), and sulfate (SO_4_^2−^)). Precipitation-weighted concentrations were calculated and used in this study [33].

At the IMPROVE site, 24-h filter samples were collected every third day [34]. Relevant to this study were the measurements of speciated mass concentrations for particulate matter (PM) with a diameter less than or equal to 2.5 μm (PM_2.5_): water-soluble ions (SO_4_^2−^, NO_3_^−^, and Cl^−^), elements (aluminum (Al), arsenic (As), bromine (Br), calcium (Ca), chromium (Cr), cupper (Cu), iron (Fe), lead (Pb), Na, magnesium (Mg), manganese (Mn), nickel (Ni), phosphorus (P), potassium (K), rubidium (Rb), selenium (Se), silicon (Si), sodium (Na), strontium (Sr), titanium (Ti), vanadium (V), zinc (Zn), and zirconium (Zr)), three fractions of elemental carbon (EC1, EC2, and EC3), and four fractions of organic carbon (OC1, OC2, OC3, and OC4). This study also used mass concentration data for fine soil and PM with diameters less than or equal to 10 μm (PM_10_). The difference between PM_10_ and PM_2.5_ is referred to as coarse particulate matter (PM_coarse_).

The water-soluble ions in the PM_2.5_ fraction were measured by ion chromatography, while the elemental mass fraction was measured by either X-ray fluorescence or particle-induced X-ray emission. The seven carbon fractions mentioned above were measured using the IMPROVE’s thermal optical reflectance (TOR) method [35,36]. The OC fractions were categorized as volatiles (OC1 volatilizes at 140 °C), semi-volatiles (OC2 at 280 °C), and non-volatiles (OC3 at 480 °C and OC4 at 580 °C) [35]. The EC fractions were classified as char-EC (EC1 at 580 °C), and soot-EC (740 °C (EC2) and 840 °C (EC3)) [35]. The char fraction represents smoldering conditions common during biomass burning and residential coal combustion, and EC2 and EC3 are emitted during flaming conditions prevalent during vehicle exhaust and coal combustion [37]. Additional details of the IMPROVE sampling protocols can be found in Chow et al. [38] and Solomon et al. [39]. Monthly averages, standard deviations, and number of samples for aerosol (IMPROVE) and precipitation chemistry (NADP) data are included in the Supplementary Information (SI) file (IMPROVE = Table S1 and NADP = Table S2). The method detection limits (MDL) for both datasets are included in Tables S3 and S4 for IMPROVE and NADP, respectively.

### 2.3. Metereological Data

Meteorological data at the Everglades NP site (25.3900° N 80.6800° W) for precipitation accumulation and temperature (minimum and maximum) were obtained from the NADP site. Wind speed and average temperature were obtained from the EPA air quality system (AQS) database [40]. Planetary boundary layer height (PBLH) data were obtained from the modern era-retrospective analysis for research and applications (MERRA-2) model with 0.5° × 0.625° spatial resolution [41]. Monthly area-averaged specific humidity (1° × 1° spatial resolution) and soil moisture (0–10 cm with 0.25° × 0.25° spatial resolution) were obtained from the global land data assimilation system (GLDAS) [42]. Cloud fraction data were retrieved from the moderate resolution imaging spectroradiometer (MODIS) on the Aqua platform and downloaded using NASA GIOVANNI [43].

### 2.4. Calculations

#### 2.4.1. Positive Matrix Factorization

Positive matrix factorization (PMF) modeling was carried out using the U.S. EPA PMF version 5A. Positive matrix factorization has been widely implemented to conduct source apportionment of PM_2.5_ [44–46]. The model was applied only to the EPA IMPROVE data (n_total_ = 658) to determine sources and evaluate corresponding contributions impacting the sampling site. The PMF model was not applied to the precipitation chemistry data as the NADP dataset had fewer species (NH_4_^+^, Ca^2+^, Cl^−^, Mg^2+^, NO_3_^−^, K^+^, Na^+^, and SO_4_^2−^), reducing the number of possible sources that can be identified; keeping the native precipitation species rather than PMF source factors allows for better intercomparison with other studies reporting the same water-soluble ion concentrations. Thirty-two species (Al, As, Br, Ca, EC1, EC2, EC3, OC1, OC2, OC3, OC4, Cl, Cr, Cu, Fe, Pb, Mg, Mn, Ni, NO_3_^−^, P, K, Rb, Se, Si, Na, Sr, SO_4_^2−^, Ti, V, Zn, and Zr) were included in the analysis. Each species was categorized depending on the signal to noise ratio (S/N). Species were classified as “Bad” if the S/N ratio was less than 0.5, “Weak” if the S/N ratio was greater than 0.5 but less than 1, and “Strong” if the value was greater than 1 [47]. Species categorized as “Bad” (e.g., EC3, OC1, Rb, Se, and Zr) were excluded from further analysis. To minimize the effect of PM_2.5_ on the model’s results, it was added as a “Total Variable” and categorized as a “weak” species [48]. Based on the method of Norris, Duvall, Brown, and Bai [47], missing concentration values were replaced with the species-specific median, samples below the method detection limit (MDL) were substituted by half of the MDL, and the uncertainty was 5/6 of the MDL. An additional 10% uncertainty was added to account for unconsidered errors for all species. Bootstrapping (BS), displacement (DISP), and bootstrapping with displacement (BS-DISP) were employed to assess the uncertainty associated with the model. To qualify a run as a successful mapping, the BS used 100 resamples with a threshold value of 0.6 for the correlation coefficient (r). The BS-DISP results further ensured that the solution is most likely a global minimum as the results did not show a significant change in the *Q* values while running DISP and BS-DISP tests. Finally, results were evaluated for the maximum change in the sum-of-squares (dQ_max_). A dQ_max_ value of 4 was chosen as it provides robust model outcomes with the smallest error [47].

#### 2.4.2. Weight Concentration Weighted Trajectory

A concentration weighted trajectory (CWT) model was used to determine the spatial distribution and transport pathways of potential PM_2.5_ sources (e.g., [49]). This approach assigns a weighted concentration to each grid cell obtained by averaging sample concentrations with associated trajectories crossing each grid cell. In order to account for uncertainty, a weighing function was applied to the CWT, which is referred to as weight concentration weighted trajectory (WCWT) [50–52]. Weight concentration weighted trajectory maps were developed using the GIS-based software TrajStat [53]. The model used a domain of 160° W to 90° E longitude and 6° N to 90° N latitude with grid cell size 0.5° × 0.5° and incorporated back-trajectories (96 h) from the hybrid single-particle Lagrangian integrated trajectory (HYSPLIT) model [54,55]. Based on previous CWT and potential source contribution function (PSCF) modeling efforts (e.g., [49,52,56,57]), the back trajectories’ ending height was set as 500 m above ground level at Everglades NP. Using the “model vertical velocity” method from the National Centers for Environmental Prediction/National Center for Atmospheric Research (NCEP/NCAR) reanalysis data, trajectories were obtained every 6 h. Seasonal WCWT maps from 2013 to 2018 are provided in Figures S1–S6 of the SI.

## 3. Results and Discussion

### 3.1. Meteorological Profile

**Figure 2.**
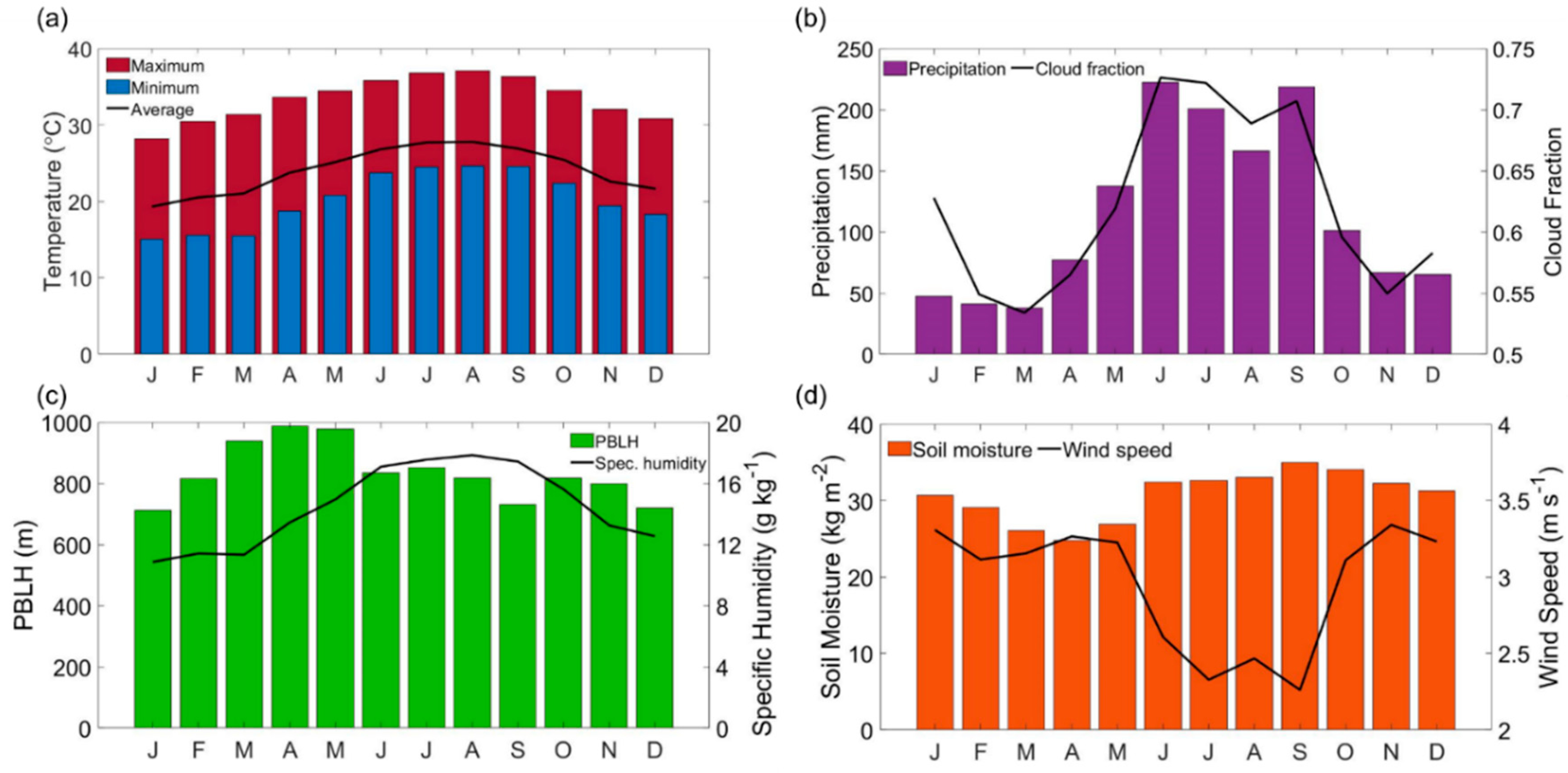
Monthly profiles of (**a**) maximum/minimum temperature (NADP) and average temperature (Environmental Protection Agency (EPA) air quality system (AQS)), (**b**) precipitation (NADP; bars) and cloud fraction (moderate resolution imaging spectroradiometer (MODIS)-Aqua; curve), (**c**) planetary boundary layer height (planetary boundary layer height (PBLH)-modern era-retrospective analysis for research and applications (MERRA-2); bars) and specific humidity (global land data assimilation system (GLDAS); curve), and (**d**) soil moisture 0–10 cm (GLDAS; bars) and wind speed (EPA AQS; curve).

The section aimed to provide an overview of the meteorological conditions at the studied region (Figure 2). Florida has a subtropical climate with mild winters and hot summers. The summer months extend from June to August (JJA) with little variability in the maximum temperature. The average temperature for the period between 2013 and 2018 was 24.0 ± 3.7 °C with a maximum temperature reported in August of 37.1 ± 2.0 °C (Figure 2a). Minimum temperatures during January, February, and March remained at 15.4 ± 4.1 °C (Figure 2a). Precipitation occurred throughout the year, but primarily between May and October (Figure 2b). Maximum precipitation values were recorded in June (223 ± 71 mm), with the minimum value registered in March (38 ± 71 mm). Higher cloud fractions followed the same trend as the monthly precipitation profile and presented a maximum value in June (0.7 ± 0.07; Figure 2b). Specific humidity values increased in conjunction with the precipitation, with the maximum value recorded in August (17.8 ± 0.48 g kg^−1^; Figure 2c). Low variability was observed in the PBLH profile throughout the year with a peak during April (989 ± 59 m), a minimum in December (711 ± 63 m), and an annual mean of 823 ± 92 m (Figure 2c). Low monthly variability in soil moisture may be related to precipitation occurring throughout the year (Figure 2d) and the fact that the site is located over a shallow aquifer [58]. The average soil moisture was 30.7 ± 3.67 kg m^−2^ for the studied period. Wind speed exhibited higher values during the months with the lowest precipitation, with a mean annual value of 2.95 ± 1.1 m s^−1^ (Figure 2d). The likelihood of enhanced local dust emissions during periods of higher winds is reduced owing to appreciable soil moisture; this contrasts to drier areas like Central California where there are enhanced dust emissions due to the combination of low soil moisture and increases in wind speed [9].

### 3.2. Sources of PM_2.5_

**Figure 3.**
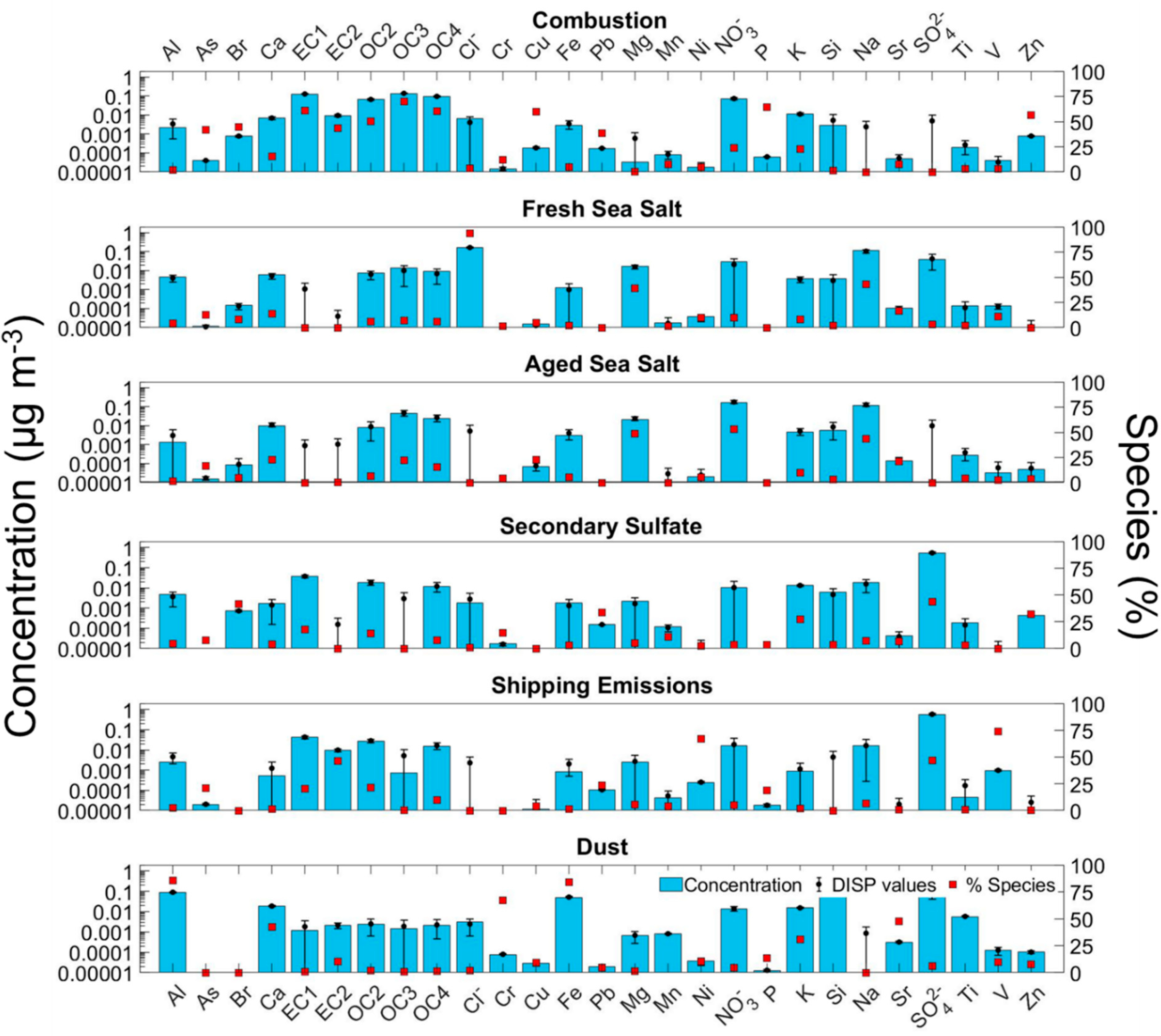
Positive matrix factorization (PMF) analysis using EPA IMPROVE data from Everglades National Park (NP). Blue bars represent species concentrations; error bars show the maximum and minimum values and black markers represent the average displacement (DISP) values. Red markers show the percent contribution from a particular source factor to each species’ overall concentration.

**Figure 4.**
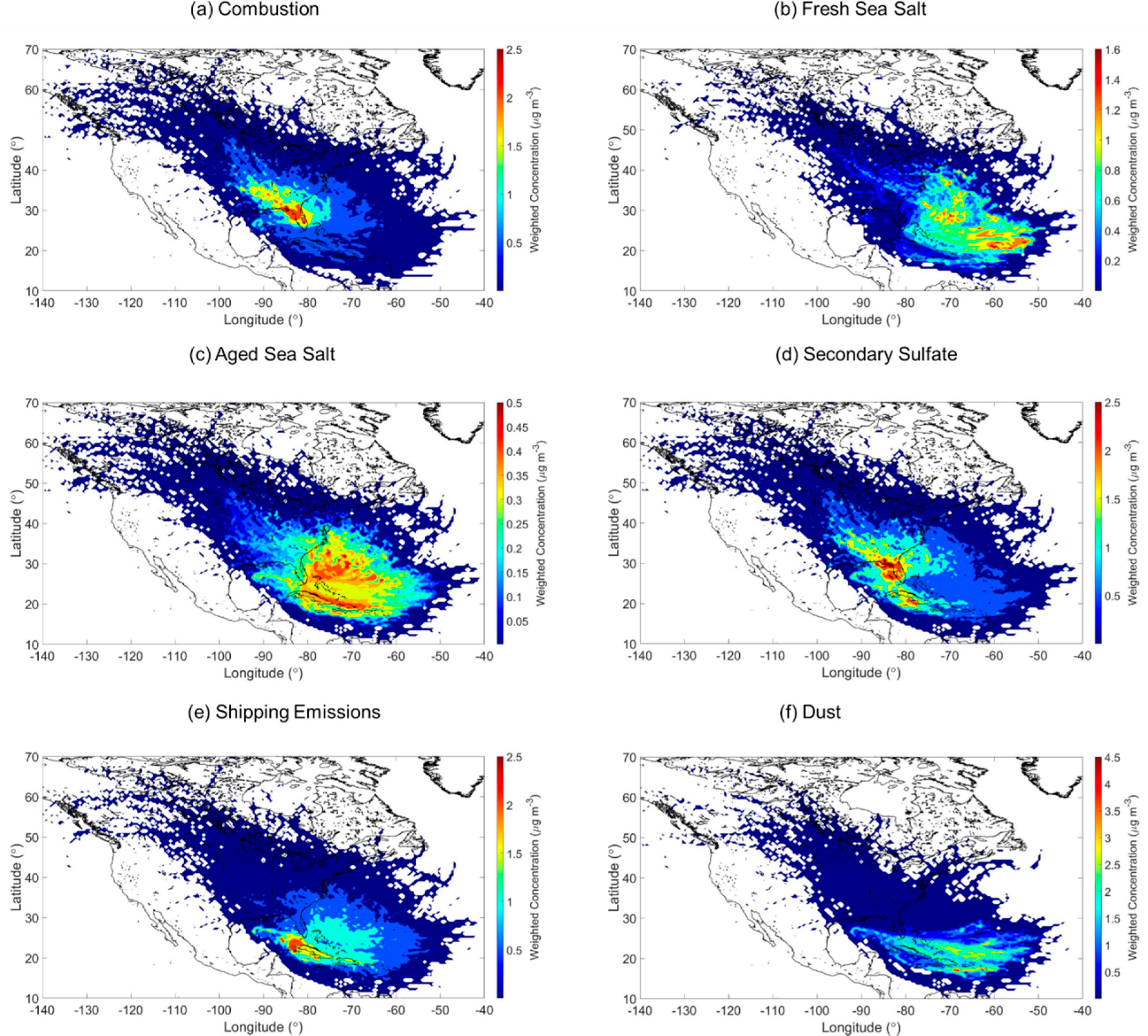
Annual mean weight concentration weighted trajectory (WCWT) maps of the six PMF source factors: (**a**) Combustion (**b**) Fresh Sea Salt (**c**) Aged Sea Salt (**d**) Secondary Sulfate (**e**) Shipping Emissions (**f**) Dust. Seasonal maps for each source factor are shown in Figures S1–S6.

A PMF model based on the elemental and inorganic ion data was used to identify potential emission sources impacting the study area. Several solutions were evaluated to converge on the final solution that has six sources, defined as follows based on their chemical characteristics: (i) combustion; (ii) fresh sea salt; (iii) aged sea salt; (iv) secondary sulfate; (v) shipping emissions; and (vi) dust. The mass concentration of different species and the percent of their mass accounted for by a specific source are shown in Figure 3. The optimal solution was chosen considering the most meaningful physical results, the lowest Q_robust_, the Q_true_/Q_expected_ ratio closest to 1, and DISP swap values equal to zero. The concentration and percent of species profiles for the preliminary PMF solutions not used as the final solution are shown in Figures S7–S10. The summary of the PMF model solutions and associated statistics are shown in Tables S5–S6. A mean annual WCWT map is shown in Figure 4 for each of the six factors identified with the PMF model. The results and discussion are included in the sections below.

#### 3.2.1. Combustion

The first factor, defined as combustion, was identified as having influence from biomass burning, vehicle emissions, industrial activities, and fuel/oil sources. Combustion accounts for 17.0% of the total PM_2.5_. Dominant constituents influenced by this source factor include the following, with percent values being the fraction of that constituent accounted for by this source: OC3 (69.7%), P (64.4%), EC1 (60.9%), OC4 (60.2%), Cu (59.6%), Zn (56.7%), OC2 (50.5%), Br (44.9%), EC2 (43.6%), As (41.8%), Pb (38.7%), NO_3_^−^ (23.7%), and K (22.5%). There were more minor contributions from this source to Ca (15.4%), Cr (12.3%), Sr (7.7%), Mn (7.5%), Ni (4.8%), Fe (4.6%), Cl (3.7%), V (3.1%), Ti (3.0%), Al (2.1%), Si (1.4%), and Mg (0.1%).

Biomass burning has been shown in past work to be an especially large contributor to EC1, P, OC2, OC3, and OC4 [59], which are prominent in this source factor. Combustion associated with either gasoline, diesel, and oil can be traced to concentrations of the OC and EC fractions and various other tracer species [60]. For example, high concentrations of OC3 and OC4 are linked to gasoline emissions as the primary source of combustion, while diesel emissions are characterized by high contributions of EC1. Oil combustion is characterized by enhanced contributions from V, and Ni [60,61]. Residual oil and pulverized coal combustion tracers include As, Cu, and NO_3_^−^ [62], while motor oil additives also include Zn and Ca [63]. Zinc and Cu are prominent species in the combustion source factor and have been shown to be pronounced in vehicular emissions [64]. Incinerating activities located in the counties surrounding the sampling site [65] likely contributed to OC1, OC4, EC1, K, Pb, Si, and Zn [66].

**Figure 5.**
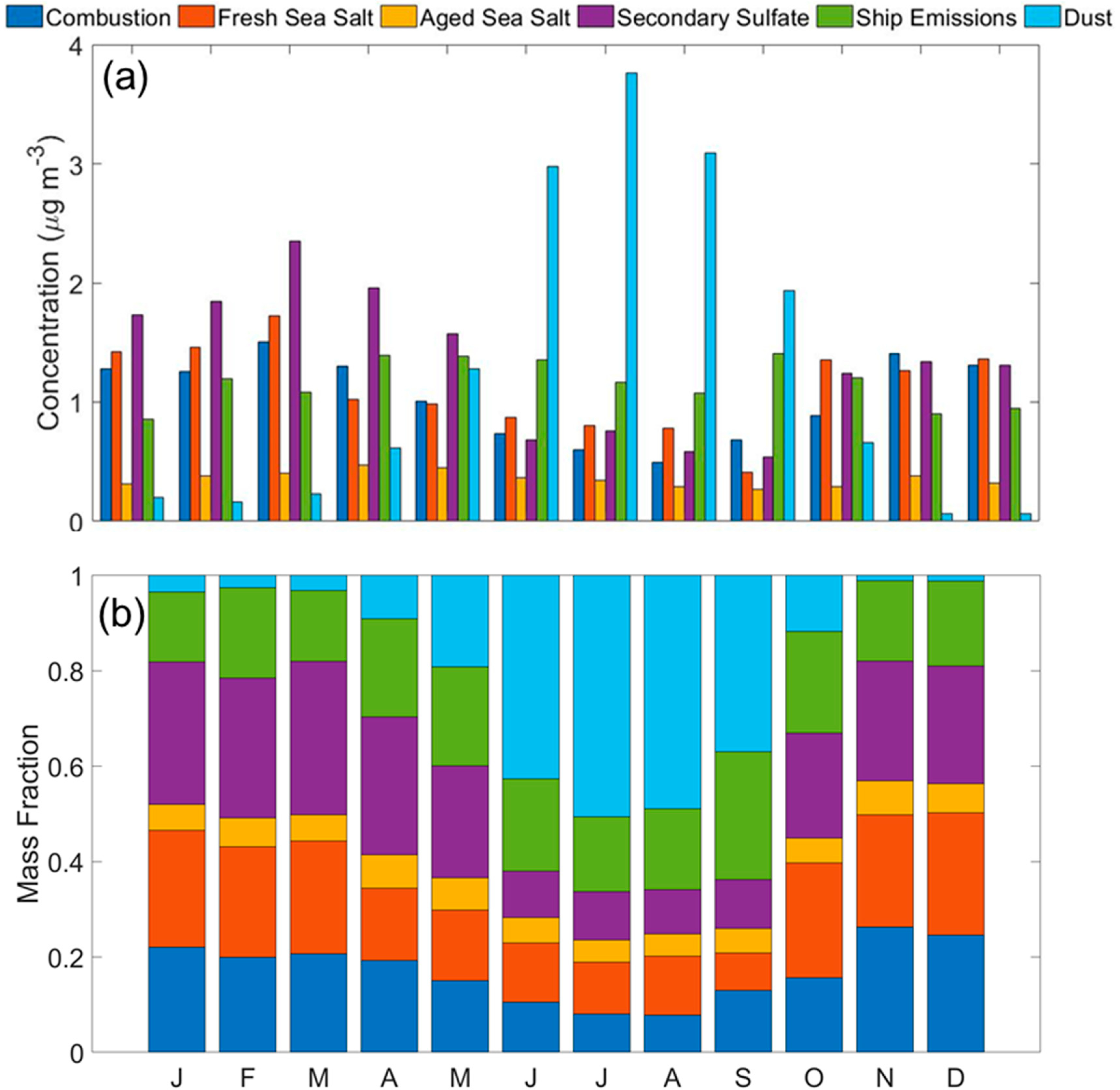
(**a**) Monthly mean aerosol concentrations and (**b**) fractional contribution for each of the six PMF source factors.

The combustion factor’s monthly profile (Figure 5a) shows higher concentrations from October to May (0.89–1.51 μg m^−3^). The relative contribution remained relatively stable throughout the year with an average value of 0.18 ± 0.06 (Figure 5b). As emissions associated with vehicular traffic, industrial activity, and fuel/oil combustion do not necessarily exhibit strong seasonal changes, other factors can explain the monthly profile. For example, the period between October and May features reduced precipitation values and thus less aerosol removal. In addition, those months coincide with the official sugarcane preharvesting burning season (mid-late October to late March) [27] and natural fires during the dry season (April and May) [67]. The WCWT map in Figure 4a shows that the major sources of combustion were over the Florida panhandle and its coast, with additional contributions from fires in the neighboring states [68–70]. Seasonal WCWT maps show higher concentrations over the Florida panhandle during SON, DJF, and MAM (Figure S1), in accordance with agricultural and natural fires reported during these months. Interestingly, the WCWT maps revealed that the influence of long-range transport of combustion emissions from fires in areas such as Mexico [15], especially the Yucatan [16], and parts of Central America [71] were not influential relative to local and regional sources.

#### 3.2.2. Fresh and Aged Sea Salt

The fresh and aged sea salt factors represent 12.6% and 6.6% of the total PM_2.5_, respectively. The fresh sea salt factor was characterized by the high contribution to Cl^−^ (93.5%), Na (43.1%), and Mg (39.3%). The main differentiating characteristic between the fresh sea salt and the aged sea salt factor was the replacement of Cl^−^ by NO_3_^−^ owing to sea salt’s chemical reactivity with acids [72,73]. More specifically, the aged sea salt factor contributed 43.5% to Na, 48.8% to Mg, 53.1% to NO_3_^−^, and 0% to Cl^−^. Chloride depletion occurs due to the reaction between sodium chloride (NaCl) and acids such as sulfuric and nitric acids (H_2_SO_4_ and HNO_3_) to form sodium nitrate (NaNO_3_) and sodium sulfate (Na_2_SO_4_) [74–77], respectively; furthermore, organic acids can participate in depletion reactions as well [78–82]. Size-resolved measurements in other coastal regions have pointed to the abundance of NO_3_^–^ in the supermicrometer size range in contrast to SO_4_^2−^ [83–85].

The monthly profile (Figure 5a) for fresh sea salt shows higher values between October and March (1.26–1.72 μg m^−3^) with a peak during March revealing a higher contribution (0.30) when compared to the rest of the year (Figure 5b). As sea salt is removed efficiently via wet scavenging with a sharp vertical gradient decreasing with altitude (e.g., [86,87]), the decrease in fresh sea salt expectedly coincides with the enhancement in precipitation between April and September as was also seen for the previously discussed combustion factor. The fresh sea salt WCWT analysis highlights higher mass concentrations associated with trajectories from the east and over the Atlantic Ocean (Figure 4b). The aged sea salt monthly profile was relatively more stable throughout the year (0.36 ± 0.06 μg m^−3^), with slightly higher levels between March–May (0.40–0.47 μg m^−3^). This coincides with the months exhibiting the highest cumulative mass concentrations from three PMF source factors known to emit acids, including combustion, shipping emissions, and secondary SO_4_^2−^. In contrast to the fresh sea salt, aged sea salt concentrations were located closer to the coast of Florida and over the Caribbean Sea (Figure 4c), which, as will be shown, was similar to maps for shipping emissions and secondary SO_4_^2−^. No apparent seasonal difference was observed for the fresh sea salt WCWT maps (Figure S2), while the seasonal aged sea salt WCWT maps (Figure S3) show a higher concentration during SON over the Atlantic Ocean north off the coast of Florida.

#### 3.2.3. Secondary Sulfate

The next factor, identified as secondary SO_4_^2−^, was the dominant factor accounting for 23.0% of the total PM_2.5_. This factor contributed the most to SO_4_^2−^ (43.6%), which is secondarily produced via gas-to-particle conversion processes from its main precursor SO_2_; however, this factor includes other important species that can be secondarily produced as well, such as secondary organic aerosol (SOA) represented by OC2 (14.2%). This factor also included contributions to Br (41.8%), Pb (33.6%), Zn (32.3%), K (27.0%), EC1 (17.9%), Cr (14.6%), Mn (10.9%), As (7.7%), OC4 (7.5%), Na (7.1%), Sr (6.4%), Mg (4.9%), Al (4.4%), Ca (4.0%), P (3.7%), NO_3_^−^ (3.4%), Si (3.3%), Ti (2.9%), Fe (2.9%), Ni (2.4%), and Cl (1.0%). As SO_4_^2−^ was most pronounced in this factor, it is worth noting that it can be secondarily produced from ocean emissions of dimethylsulfide (DMS) [88], while also originating from both local/regional sources and long-range transport of anthropogenic pollution. The contribution of the various elements to this factor is suggestive of their coemissions with SO_2_. For instance, Pb and Zn are coemitted with SO_4_^2−^ precursors from municipal solid waste incinerators [89] located 80 km northeast of the sampling site [65]. Other sources coemitting elements along with SO_4_^2−^ precursors include: emissions from gasoline vehicles (e.g., Br, EC1, and OC2) [90,91]; Cr electroplater and anodizer facilities (e.g., Cr) [65,92]; and sugarcane preharvest burning (e.g., K, EC1, Zn, and Br) [27,56,93].

The monthly profile and fractional contribution of secondary SO_4_^2−^ (Figure 5a,b) follow the same trend as most other factors discussed already, with higher values between October and May (1.24–2.35 μg m^−3^), coincident with reduced precipitation scavenging. Secondary SO_4_^2−^ relies on production via aqueous-phase chemistry (e.g., [94]), which benefits in the study region from fairly high year-round values of cloud fraction and humidity (Figure 2). The WCWT spatial results for secondary SO_4_^2−^ show more considerable influence from marine regions as compared to the combustion factor (Figure 4d). The same can be observed in the seasonal WCWT maps, where the hotspots were concentrated in the area surrounding the southern coast of Florida. There was not a notable difference in the seasonal WCWT maps for this factor (Figure S4).

#### 3.2.4. Shipping Emissions

The shipping emissions factor represents 20.2% of the total PM_2.5_. The high contributions to V (73.7%), Ni (67.1%), SO_4_^2−^ (46.9%), EC2 (46.4%), Pb (23.4%), OC2 (21.6%), As (21.3%), EC1 (20.5%), P (18.8%), and OC4 (9.9%) confirmed the primary source of this factor as they are commonly found in low-cost residual oil used as ship fuel [95–99]. The identity of this source was further confirmed by the calculated V:Ni ratio of 3.9, which fits in the range (2.5–5) suggested by Pandolfi et al. [100] as a marker for shipping emissions.

The PM_2.5_ monthly profile and fractional contribution for shipping emissions in Figure 5a,b exhibited a bimodal distribution with peaks in April-June (1.35–1.39 μg m^−3^) and September-October (1.20–1.40 μg m^−3^). Compared to the other five factors, this factor showed the least variability among all the aerosol sources. The shipping emissions WCWT map (Figure 4e) shows that substantial contributions come expectedly from marine areas, especially along the Florida, Cuba, Haiti, and the Dominican Republic coasts. Areas of high ship density coincide with high concentrations in the WCWT map. Seasonal WCWT maps do not show notable differences (Figure S5).

#### 3.2.5. Dust

The dust source factor constitutes 20.6% of the total PM_2.5_. The high contribution of this source factor to crustal elements like Si (90.5%), Ti (86.9%), Al (85.5%), Fe (83.9%), Mn (75.9%), Cr (67.3%), Sr (47.5%), Ca (42.4%), and K (31.0%) clearly defined this factor as dust [14,32,101,102]. Additional support for this source being linked to dust was the high concentrations and fractional contribution observed during JJA, ranging from 2.98 to 3.76 μg m^−3^ and 0.51 to 0.65, respectively (Figure 5a,b), which is when long-range transport of dust commonly occurs from Africa towards the Caribbean region (e.g., [101,103,104]) and Miami [105–107]. It should be noted that these values are lower compared to the long-term means reported in previous studies [108], owing largely to their inclusion of particles with diameters above 2.5 μm [109,110]. The influence of local dust is likely low as the highest dust concentrations coincided with the months with the highest precipitation and soil moisture (Figure 2b,d).

The summer dust WCWT map (Figure 4f) confirmed long-range transport of dust from Northern Africa with the highest concentration observed during this season (Figure S6), consistent with previous studies [14,105,108,111–113]. The seasonal WCWT map (Figure S6) shows a clear seasonal difference both in the trajectory paths and in absolute dust concentrations. The summertime (JJA) results show the highest concentrations approaching from the east (Northern Africa).

### 3.3. Precipiation Chemistry Profile

#### 3.3.1. Monthly Profile

**Figure 6.**
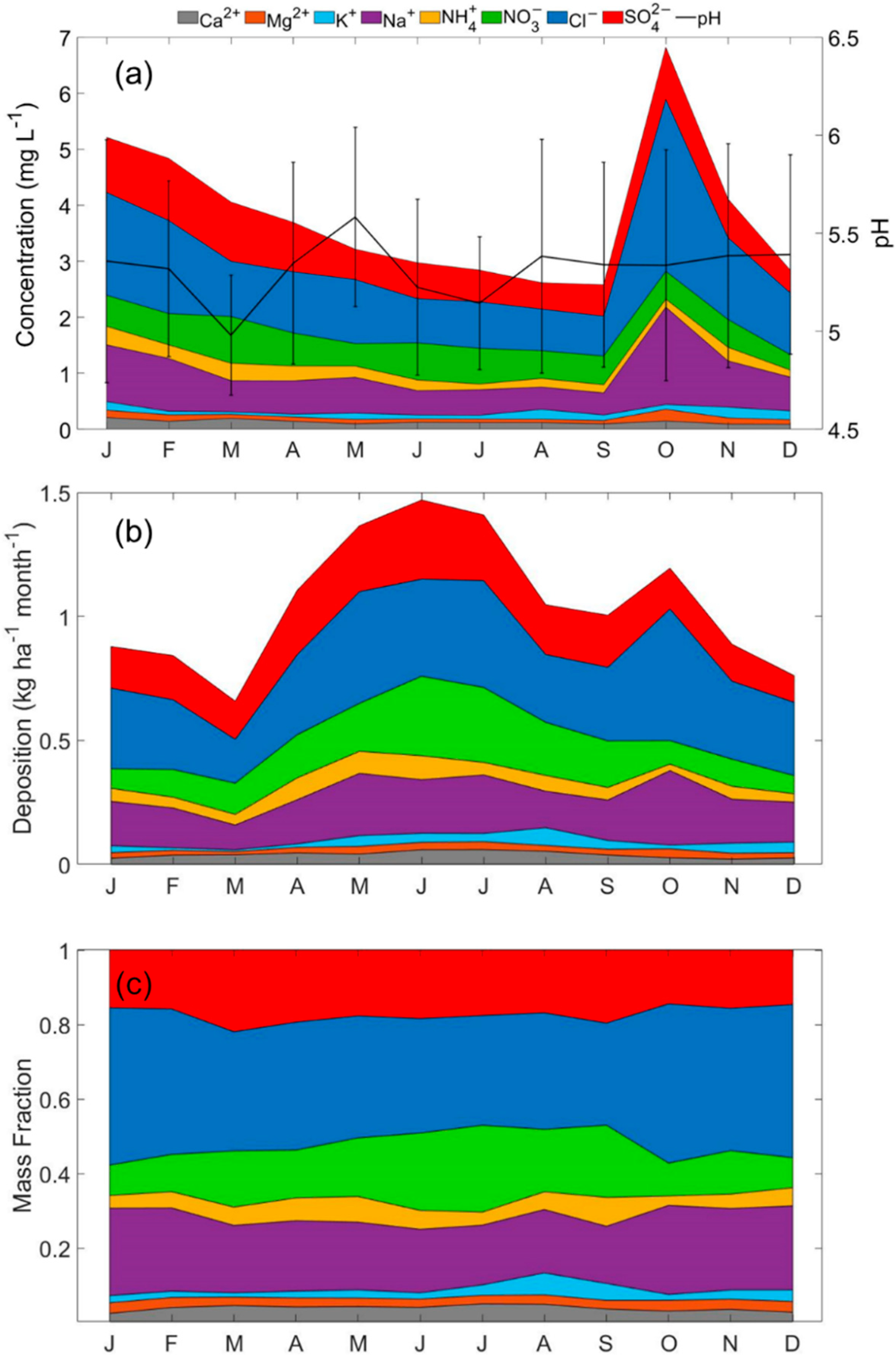
Monthly averaged (**a**) precipitation-weighted ion concentrations and pH, (**b**) wet deposition fluxes, and (**c**) mass fractions.

The monthly averaged profiles for speciated precipitation data (concentration, deposition flux, and mass fraction) are shown in Figure 6. Wet deposition pH is of great importance in terms of the negative impacts of acid rain. A threshold value of 5.6 is often used to distinguish acid rain (pH < 5.6), symbolic of deionized water in equilibrium with CO_2_ (e.g., [114]). However, wet deposition samples contain influence from the original CCN/IN and other gases and aerosols that were scavenged and thus were never pure water. The mean annual pH was 5.32 ± 0.51. Monthly averaged pH values ranged from as low as 4.98 ± 0.31 during March to as high as 5.58 ± 0.46 in May. For contrast, the mean pH was 5.05 at Everglades NP between September 1992 and October 1993 [115]. Documented values for other regions include the following with the caveat that time periods of analysis varied: 5.33–5.51 in Central California [9], 4.20–7.39 in India [116], 4.22–5.68 in Southeastern Brazil [117], 3.52–6.28 in South China [118], 5.71–7.11 in the Southeast Tibetan Plateau [119], and 3.8–6.8 in Eastern France [120]. Regions with the highest reported pH values typically were characterized by having high dust influence and thus carbonate bases such as CaCO_3_ ([10]), including in Southwestern Iran (up to 7.38) [121] and Northwestern Iran (up to 8.6) [122], and the Central Himalayas (up to 6.50) [123]. In this work, the wet deposition pH did not exhibit a pH peak during the months with most dust influence in the summer. This was an intriguing result suggestive of transported dust not being a factor in promoting higher pH values in the region’s rainfall.

The total concentration sum of the eight speciated ions was elevated between October and April (Figure 6a), coinciding with the months experiencing the least rain and highest concentrations of most PMF aerosol source factors except shipping emissions and dust. Summer months (JJA) associated with high levels of dust did not show high concentrations of crustal species like Ca^2+^ and Mg^2+^, as observed in the IMPROVE data. This again highlights that the summertime dust season does not play a major role in impacting wet deposition chemistry on a volume-weighted basis. In contrast to their relatively low influence in PM_2.5_, sea salt constituents (i.e., Cl^−^ and Na^+^) exhibited the highest concentrations of all ions studied. Sea salt particles are an important source of CCN due to their large size and hygroscopicity, which allows for easier droplet activation as compared to other aerosol types [9,124–126]. Higher concentrations of Cl^−^, SO_4_^2−^, and Na^+^ in precipitation as compared to PM_2.5_ may be partly explained by how the wet deposition data include influence from particles of any size participating in cloud processes or subcloud scavenging. In contrast, the aerosol data were limited to ≤2.5 μm. Sea salt typically ranges from 0.01 and 10 μm [127], with the majority of the mass accounted for by those particles with diameters above 2.5 μm.

An interesting result was the unusually high October levels of Cl^−^ and Na^+^ since sea salt is not expected to show such an increase in a particular month. We also examined the available data back to 1980 and did not observe unusual concentrations as the ones observed during this period. Further analysis of the dataset (2013–2018) showed elevated Cl^−^ and Na^+^ in mid-October 2015 and 2016 (Cl^−^: 4.3–16.1 mg L^−1^ vs. 0.6–2.2 mg L^−1^ for other periods; Na^+^: 2.5–9.3 mg L^−1^ vs. 0.4–1.3 mg L^−1^ for other periods; Table S7). Even though the concentrations were significantly higher during these few samples (5 out of 14 total for October), the Cl^−^:Na^+^ ratio for these samples (1.70–1.97) was still close to that of pure seawater (Table S7). While a few periods of stronger sea salt emissions may have been likely, another possibility is inspired by previous studies have reported that biomass burning could be an important source of Na^+^ and Cl^−^ (e.g., [128–132]). This has been discussed already to be an important contributor to the regional aerosol pollution between October and March. To probe deeper, images from the Navy Aerosol Analysis and Prediction System (NAAPS; description in Section S1 of SI) were used to detect the possible presence of smoke during the days with high Na^+^ and Cl^−^ concentrations (Figures S10–S16). The NAAPS images did show evidence of smoke for the five week-long periods corresponding to the high Cl^−^ and Na^+^ data points; however, aerosol concentrations of the biomass burning marker K and PMF combustion factor did not show any remarkable enhancement during the time of the five NADP samples of interest (Table S7). Future research can investigate as to whether emissions of Cl^−^ and Na^+^ from regional combustion sources reside in sizes exceeding 2.5 μm, especially during smoke periods, which can help reconcile the difference between the aerosol and wet deposition results for October.

In comparison to the precipitation ion concentrations, the opposite monthly trend was observed for the average monthly deposition fluxes, where higher values were observed during the high precipitation months (April–October; Figure 6b). The highest deposition flux value (1.05 ± 0.26 kg ha^−1^ month^−1^) was observed during June when precipitation reached its maximum value. The highest mean annual deposition fluxes were attributed to Cl^−^ (0.53 kg ha^−1^ month^−1^), NO_3_^−^ (0.32 kg ha^−1^ month^−1^), SO_4_^2−^ (0.32 kg ha^−1^ month^−1^), and Na^+^ (0.30 kg ha^−1^ month^−1^). On the other hand, Ca^2+^, K^+^, and Mg^2+^ showed lower deposition fluxes, each being less than 0.1 kg ha^−1^ month^−1^. However, deposition fluxes for all three of those ions showed an increase during the summer months compared to other times of the year due most likely to dust influence.

While there were seasonal differences in the concentrations and deposition fluxes of the eight ions, their relative amounts are of significance to gain insight about the relative influence of various sources rather than absolute amounts of constituents. The monthly mass fraction profile was dominated by Cl^−^ (0.27–0.43) and followed by Na^+^ (0.15–0.24), NO_3_^−^ (0.08–0.23), and SO_4_^2−^ (0.14–0.22; Figure 6c). As observed for other coastal sites, the proximity of the sampling station to the coast explains the high relative contribution of sea salt species like Cl^−^ and Na^+^ (e.g., [133,134]). Grimshaw and Dolske [115] reported decades earlier for the same study region that, based on calculations involving Na ratios, 34% of cations and 65% of anions in wet deposition were of oceanic origin. Sea salt constituents peaked in their mass fractions between October and February.

Chloride-to-sodium ratios are commonly used as a marker for the presence of sea salt and possible Cl^−^ depletion [135,136]. Marine aerosols over the ocean have a Cl^−^:Na^+^ mass concentration ratio for seawater of 1.8 [136,137]. The ratio values for precipitation samples should be carefully interpreted, as uncertainties may arise from this calculation (e.g., [138,139]). For instance, HCl produced after Cl^−^ depletion of sea salt has a longer atmospheric lifetime versus deposition in contrast to its parent aerosol [140]. Little variability was observed between the monthly mass concentration ratios of Cl^−^:Na^+^, with values ranging from 1.74 to 1.84. These results were supportive of a lack of significant sea salt reactivity with inorganic and organic acids [82], which would reduce the ratio. This result was consistent with the PMF analysis showing that fresh sea salt was nearly twice as abundant as aged sea salt. Furthermore, the ratios did not significantly exceed 1.8, pointing to reduced influence from non-salt sources of Cl^−^, such as from coal fired generating facilities and waste incineration, in contrast to wet deposition data from New York [141]. Strong deviations below a ratio of 1.8 could be linked to non-salt sources of Na^+^, such as soil, but that was not the case in this study, consistent with a general lack of influence from crustal species compared to sea salt. Junge and Werby [142] reported on the variability of Cl^−^:Na^+^ mass concentration ratios between the coast and inland U.S. between 1955 and 1956. Ratio values along the coast were lower than those for seawater, ranging from 1.06 to 1.75. However, Cl^−^:Na^+^ ratios drastically dropped farther inland as an indication of enhanced Na^+^ from contributions of non-salt sources (mineral dust). Therefore, it is an important result that the sea salt impacting precipitation was relatively pristine when considering the various other sources impacting the area.

High mass fractions for SO_4_^2−^ and NO_3_^−^ are suggestive of contributions from anthropogenic emissions and combustion of oil products (e.g., shipping traffic) [143] due to the proximity of urban areas and marine ports (e.g., Miami and Port Everglades). Interestingly, SO_4_^2−^ mass fractions were highest between March-April and September (0.20–0.22), whereas NO_3_^−^ fractions were highest in June–July (0.21–0.23). Additional contributions to the SO_4_^2−^ mass fraction may be attributed to sea salt and secondary production from DMS emissions over the ocean. Additionally, another species commonly linked to SO_4_^2−^ and NO_3_^−^ anions owing to secondary salt formation is NH_4_^+^. Ammonium showed a monthly profile (range = 0.03–0.08) more accordant with SO_4_^2−^, suggestive of potential secondary salt formation between those two species (e.g., ammonium sulfate/bisulfate). Mass size distributions of NO_3_^−^ commonly indicate significant contributions to the coarse aerosol fraction. Thus, its different monthly profile may be linked to a summertime association with coarse dust, unlike secondarily produced salts.

The least abundant species (Ca^2+^, Mg^2+^, and K^+^) had mass fractions ranging from 0.01 to 0.06 throughout the year. All three species peaked at different times of the year, pointing to different influential sources. For instance, Ca^2+^ peaks in July-August (0.05) and is thus temporally coincident with the dust season, whereas Mg^2+^ peaks between October and February (0.03), which overlaps with when the sea salt constituent mass fractions peak. Lastly, K^+^ peaked in August and September (0.05–0.06), which was likely linked to dust.

#### 3.3.2. Interrelationships

Another way to examine the precipitation chemistry data is to understand the relationship between the species present in precipitation. Table 1 reports on the interrelationships between the NADP species in the form of a correlation matrix showing values of the Pearson’s correlation coefficient (r) significant at the 95% confidence level (i.e., *p*-value < 0.05). The strength of the relationship can be categorized as follows for the absolute r value: r = 0–0.2: weak; r = 0.21–0.4: moderately weak; r = 0.41–0.6: moderate; r = 0.61–0.8: moderately strong; and r ≥ 0.81: strongly correlated [144]. Interestingly, all the species concentrations in Table 1 exhibited statistically significant correlations between each other (i.e., *p*-value < 0.05), but a few were notable. Species that were positively correlated with pH included Ca^2+^ (r = 0.31; moderately weak), NO_3_^−^ (r = 0.07; weak), NH_4_^+^ (r = 0.51; moderate), and K^+^ (r = 0.45; moderate). The three cations were commonly associated with dust and smoke. Precipitation pH showed a moderate weak negative correlation with the acidic species SO_4_^2−^ (r = −0.29), consistent with past work in the study region [145]. The lowest precipitation pH value coincided with the highest SO_4_^2−^ concentration, reported in March. Even though high concentrations of SO_4_^2−^ were recorded throughout the year, it is possible that these do not have a larger impact on the precipitation pH due to the neutralizing effect of NH_4_^+^ [115,146] and Ca^2+^ [147].

The strongest correlation among all of the species was between Cl^−^ and both Na^+^ (r = 1.00) and Mg^2+^ (r = 0.99), which is expected from a near-coastal location influenced by sea salt [10,83,148]. The strongest relationship for the crustal tracer Ca^2+^ was with NO_3_^−^ (r = 0.89), which suggests partitioning of NO_3_^−^ to dust, as shown in previous studies [149–151]. A moderate correlation between Ca^2+^ and SO_4_^2−^ (r = 0.41) suggests that NO_3_^−^ preferentially participates in reactions with coarse particles that serve as CCN/IN, supported by the monthly profile of mass fractions in the NADP data mentioned earlier. However, there could be potentially enhanced uptake of nitric acid (HNO_3_) in existing droplets enriched with Ca^2+^ [10,85,152]. In contrast, NO_3_^−^ was weakly correlated with sea salt constituents (Na^+^ and Cl^−^; r = 0.15–0.16), which was surprising as many studies associate NO_3_^−^ with sea salt aerosols [83–85,148]. Since fresh sea salt was more abundant in the study region than aged sea salt, it is possible that there simply was less NO_3_^−^ available to those salt particles seeding the regional clouds. Certainly, more size-resolved measurements of particles in the region can help solidify some of the speculated conclusions reached here regarding relationships between NO_3_^−^ with both sea salt and dust particles.

### 3.4. Precipitation and Aerosol Interrelationships

Concentrations of the precipitation species are related to the PMF source factor and aerosol species concentrations. The PMF and aerosol chemistry concentrations were averaged to the time resolution of NADP data to allow for a direct comparison in the form of a correlation matrix (Table 2). Notable results are as follows with the caveat that speculated relationships require more detailed measurements in future research as this analysis relies on surface measurements.

First, the major sea salt constituents in wet deposition (Na^+^, Mg^2+^, and Cl^−^) were similarly related to the same species in the aerosol samples and the PMF fresh sea salt factor. These results suggest how a directly emitted marine aerosol type similarly impacts both PM_2.5_ and wet deposition at a coastal site and show that the technique used to relate the aerosol and precipitation data offers promise to build on in future studies. Sulfate from NADP samples showed a weak positive correlation with Na^+^ (r = 0.20) and Cl^−^ (r = 0.17) in the aerosol dataset (but not Mg^2+^), which was not surprising as it is an important constituent in natural sea salt. The same sea salt constituents in wet deposition samples interestingly exhibit a weak negative relationship with fine soil in the aerosol dataset. This relationship can likely be related to the seasonal variation of these species. Sodium, Mg^2+^, and Cl^−^ in the precipitation data peak during the fall months (SON), while aerosol fine soil levels peak during the summer months (JJA). The lack of any relationship between the sea salt constituents in the NADP datasets with the aged sea salt factor from PMF analysis implied that the region’s precipitation was more representative of natural sea salt rather than sea salt that had undergone chemical reactions.

Similar to how sea salt components showed a statistically significant correlation (*p*-value < 0.05) between the two datasets, the same applies to SO_4_^2−^ owing to how the secondary SO_4_^2−^ source factor exhibited a moderately weak correlation with precipitation SO_4_^2−^ (r = 0.23). Precipitation SO_4_^2−^ also exhibited moderately weak (r = 0.30) and weak (r = 0.14) correlations with fresh sea salt and combustion PMF factors, respectively. The weak to moderately weak positive correlations for precipitation SO_4_^2−^ can be explained by how SO_4_^2−^ was present in sea salt, and it was also produced from secondary formation from both biogenic [149,153] and anthropogenic [154,155] precursor emissions. Precipitation NH_4_^+^ exhibited a weak positive correlation with only the secondary SO_4_^2−^ PMF factor (r = 0.15), consistent with ammonium sulfate formation. Since precipitation NO_3_^−^ did not exhibit any correlation with the same species in the IMPROVE dataset, this suggests that its involvement with wet deposition was based on processes unrelated to surface NO_3_^−^ formation in PM_2.5_, including its association with CCN/IN larger than 2.5 μm [84,85] and also HNO_3_ partitioning to cloud and precipitation drops aloft, as has been documented in other works [10,85,152].

There was a weak positive correlation between precipitation Ca^2+^ and both aerosol NO_3_^−^ (r = 0.17) and PM_coarse_ (r = 0.18). It was already suggested by Table 1 that NO_3_^−^ preferentially partitions to coarse dust particles (rather than sea salt), and these results support that notion. The lack of a correlation between precipitation Ca^2+^ and either fine soil or the dust PMF factor could be due to the latter two IMPROVE parameters being limited to the PM_2.5_ fraction, whereas the dust particles involved with precipitation processes are larger. An interesting hypothesis motivated by Table 2 warranting future research was that the combustion PMF factor was correlated with both precipitation Ca^2+^ and NO_3_^−^ partly because soil dust can be entrained in biomass combustion plumes (e.g., [84,156]).

The lack of other relationships in Table 2 between wet deposition species and pH with aerosol constituents points to the complexities of comparing the datasets and in the processes leading to wet deposition. This was further emphasized by how the correlation coefficients in Table 2 do not exceed a value of 0.36. A major difference is that the IMPROVE speciated data are based on PM_2.5_. In contrast, larger particles (mainly sea salt) have been shown here to be especially influential in the collected wet deposition samples. Therefore, any species associated with the coarse sea salt that served as the CCN/IN and any species taken up by existing drops (e.g., HNO_3_) would not have been similarly observed at the surface IMPROVE monitoring site. As Table 1 showed, sea salt constituents were positively correlated with all the other species in the NADP dataset. Thus, if they were all linked to the coarse sea salt CCN/IN, this would complicate and reduce the correlations reported in Table 2. Although this highlights an important limitation of comparing the two datasets, it importantly highlights the significance of sea salt and the prominence of its associated constituents in the region’s rainfall. Regarding aerosol–cloud interactions, sea salt was shown here to be an important CCN/IN year-round.

## 4. Conclusions

The analysis of long-term precipitation chemistry and aerosol data from a coastal site in the Southeast U.S. was used to gain insight into emissions sources impacting both PM_2.5_ and wet deposition. The two datasets were intercompared to gain insights into aerosol–precipitation relationships. The points below summarize the findings of this study:

- The Southern Florida coastal site was impacted by a diverse source of pollutants. The following six sources were identified in decreasing contribution to total PM_2.5_ (percentage contributions): (i) secondary sulfate (23.0%); (ii) dust (20.6%); (iii) shipping emissions (20.2%); (iv) combustion (17.0%); (v) fresh sea salt (12.6%); and (vi) aged sea salt (6.6%).
- Monthly mean precipitation pH ranges from 4.98 ± 0.31 (March) to 5.58 ± 0.51 (May). Values of pH were negatively related to the acidic anion SO_4_^2−^, whereas they were positively related to dust presence as based on the crustal tracer species Ca^2+^.
- The highest mean annual wet deposition fluxes were attributed to Cl^−^, NO_3_^−^, SO_4_^2−^, and Na+ between April and October, coinciding with months experiencing the most precipitation. Although lower in magnitude, enhanced fluxes of Ca^2+^, K^+^, and Mg^2+^ in summertime coincided with the main dust season.
- Fresh sea salt was the dominant component in the region’s precipitation, unlike surface PM_2.5_ owing partly to how the sea salt particles seeding precipitation drops likely exceed 2.5 μm. Aged sea salt was shown to be far less influential in the region’s precipitation.
- Even though dust plays a large role in PM_2.5_, it was much less influential in volume-weighted wet deposition concentrations and mass fractions as compared to sea salt.
- A weak positive association between precipitation Ca^2+^ and both aerosol NO_3_^−^ and PM_coarse_ was linked to NO_3_^−^ preferentially partitioning to coarse dust.
- Statistically significant correlations (p-value < 0.05) between related parameters indicative of dust, sea salt, and SO_4_^2−^ in the NADP and IMPROVE datasets demonstrated that the combined use of these long-term datasets could be useful for other regions to indirectly examine aerosol-precipitation interactions.

## Supplementary Material

SI

## Figures and Tables

**Table 1.** Correlation coefficient (r) matrix between concentrations of different species in wet deposition samples (NADP). Values shown are statistically significant at the 95% confidence level.

Species	pH	Ca^2+^	Mg^2+^	K^+^	Na^+^	NH_4_^+^	NO_3_^−^	Cl^−^	SO_4_^2−^

pH	1								
Ca^2+^	0.31	1							
Mg^2+^		0.33	1						
K^+^	0.45	0.16	0.35	1					
Na^+^		0.31	0.99	0.26	1				
NH_4_^+^	0.51	0.48	0.22	0.42	0.18	1			
NO_3_^−^	0.07	0.89	0.18	0.09	0.16	0.47	1		
Cl^−^		0.29	0.99	0.26	1.00	0.17	0.15	1	
SO_4_^2−^	−0.29	0.41	0.66	0.20	0.66	0.40	0.40	0.64	1

**Table 2.** Correlation coefficient (r) matrix between precipitation (NADP) and aerosol (IMPROVE) composition data. Values shown are statistically significant at the 95% confidence level. The first six aerosol parameters are concentrations of PMF source factors, while the final six at the bottom are selected IMPROVE parameters relevant to the discussion in Section 3.4. Coarse particulate matter (PM_coarse_) is calculated as particulate matter with a diameter less than or equal to 10 μm (PM_10_)-particulate matter with a diameter less than or equal to 2.5 μm (PM_2.5_).

Aerosol Parameters	Wet Deposition							
	Ca^2+^	Mg^2+^	K^+^	Na^+^	NH_4_^+^	NO_3_^−^	Cl^−^	SO_4_^2−^

**Combustion**	0.15					0.16		0.14
**Fresh Sea Salt**		0.32		0.33			0.33	0.30
**Aged Sea Salt**								
**Secondary Sulfate**			−0.14		0.15			0.23
**Shipping Emissions**		−0.17		−0.17			−0.17	
**Dust**					−0.16			−0.18
**Mg**		0.23		0.24			0.24	
**Na**		0.32		0.33			0.33	0.20
**NO_3_^−^**	0.17							
**Cl^−^**		0.35		0.36			0.35	0.17
**PM_coarse_**	0.18							
**Fine Soil**		−0.18		−0.19	−0.16		−0.19	−0.21
