# Supplementary material for: Source Apportionment of Aerosol at a Coastal Site and Relationships with Precipitation Chemistry: A Case Study over the Southeast United States
Source: Atmosphere (Basel). Author manuscript; Available in PMC 2021 Jun 30. (PMC8243544; doi:10.3390/atmos11111212)
Supplement: SI [file NIHMS1710871-supplement-SI.pdf]

Source Apportionment of Aerosol at a Coastal Site and Relationships With Precipitation Chemistry: A Case Study Over the Southeast United States

Andrea F. Corral [1,*], Hossein Dadashazar [1], Connor Stahl [1], Eva-Lou Edwards [1], Paquita Zuidema [2], Armin Sorooshian [1,3]

[1] Department of Chemical and Environmental Engineering, The University of Arizona, Tucson, AZ 85721; afcorral@arizona.edu (A.F.C.); hosseind@arizona.edu (H.D.); cstahl1@uits.arizona.edu (C.S.); evalouedwards@uits.arizona.edu (E.E.); armin@arizona.edu (A.S.)

[2] Rosenstiel School of Marine and Atmospheric Science, University of Miami, Miami, FL 33149; pzuidema@rsmas.miami.edu

[3] Department of Hydrology and Atmospheric Sciences, The University of Arizona, Tucson, AZ 85721

* Correspondence: afcorral@arizona.edu

S1. Navy Aerosol Analysis and Prediction System Description

Images from the Navy Aerosol Analysis and Prediction System (NAAPS) [1,2] were used to confirm the presence of smoke. The model relies on global meteorological fields from the Navy Global Environmental Model (NAVGEM) [3]. Long-range transport of aerosols using NAAPS has been used expansively [4–8], including for smoke detection [9–12]. The surface concentration of smoke is presented in units of μg m^{-3} at 6-hour resolution.

Table S1. Monthly mean and standard deviation for the aerosol (IMPROVE) dataset. Number of data points available is shown as "n". Lack of data is shown as "NaN".

Month	n	Al	As	Br	Ca	EC1	EC2
January	58	$1.41 \times 10^{-2} \pm 1.30 \times 10^{-2}$	$4.28 \times 10^{-4} \pm 5.12 \times 10^{-4}$	$2.62 \times 10^{-3} \pm 1.60 \times 10^{-3}$	$2.75 \times 10^{-2} \pm 1.77 \times 10^{-2}$	$2.39 \times 10^{-1} \pm 2.00 \times 10^{-1}$	$2.27 \times 10^{-2} \pm 1.39 \times 10^{-2}$
February	54	$1.49 \times 10^{-2} \pm 1.21 \times 10^{-2}$	$4.21 \times 10^{-4} \pm 2.91 \times 10^{-4}$	$2.80 \times 10^{-3} \pm 1.55 \times 10^{-3}$	$3.41 \times 10^{-2} \pm 4.28 \times 10^{-2}$	$2.82 \times 10^{-1} \pm 1.92 \times 10^{-1}$	$2.62 \times 10^{-2} \pm 1.56 \times 10^{-2}$
March	59	$2.75 \times 10^{-2} \pm 2.66 \times 10^{-2}$	$3.11 \times 10^{-4} \pm 1.88 \times 10^{-4}$	$3.21 \times 10^{-3} \pm 1.67 \times 10^{-3}$	$1.20 \times 10^{-1} \pm 2.58 \times 10^{-1}$	$3.41 \times 10^{-1} \pm 3.70 \times 10^{-1}$	$3.95 \times 10^{-2} \pm 3.42 \times 10^{-2}$
April	52	$5.67 \times 10^{-2} \pm 9.37 \times 10^{-2}$	$4.91 \times 10^{-4} \pm 3.76 \times 10^{-4}$	$2.70 \times 10^{-3} \pm 1.58 \times 10^{-3}$	$5.99 \times 10^{-2} \pm 3.86 \times 10^{-2}$	$3.25 \times 10^{-1} \pm 3.03 \times 10^{-1}$	$2.73 \times 10^{-2} \pm 1.59 \times 10^{-2}$
May	52	$1.01 \times 10^{-1} \pm 1.95 \times 10^{-1}$	$4.58 \times 10^{-4} \pm 5.07 \times 10^{-4}$	$2.33 \times 10^{-3} \pm 2.12 \times 10^{-3}$	$4.98 \times 10^{-2} \pm 4.99 \times 10^{-2}$	$2.77 \times 10^{-1} \pm 2.89 \times 10^{-1}$	$3.09 \times 10^{-2} \pm 2.79 \times 10^{-2}$
June	53	$2.55 \times 10^{-1} \pm 2.92 \times 10^{-1}$	$4.88 \times 10^{-4} \pm 6.25 \times 10^{-4}$	$1.36 \times 10^{-3} \pm 2.06 \times 10^{-3}$	$6.45 \times 10^{-2} \pm 5.71 \times 10^{-2}$	$1.81 \times 10^{-1} \pm 1.55 \times 10^{-1}$	$2.97 \times 10^{-2} \pm 1.77 \times 10^{-2}$
July	60	$3.45 \times 10^{-1} \pm 2.99 \times 10^{-1}$	$2.77 \times 10^{-4} \pm 1.84 \times 10^{-4}$	$9.71 \times 10^{-4} \pm 5.88 \times 10^{-4}$	$8.35 \times 10^{-2} \pm 6.20 \times 10^{-2}$	$1.34 \times 10^{-1} \pm 8.88 \times 10^{-2}$	$3.08 \times 10^{-2} \pm 1.79 \times 10^{-2}$
August	61	$2.52 \times 10^{-1} \pm 3.20 \times 10^{-1}$	$2.66 \times 10^{-4} \pm 1.44 \times 10^{-4}$	$8.77 \times 10^{-4} \pm 4.50 \times 10^{-4}$	$6.90 \times 10^{-2} \pm 6.97 \times 10^{-2}$	$1.23 \times 10^{-1} \pm 7.06 \times 10^{-2}$	$2.84 \times 10^{-2} \pm 2.08 \times 10^{-2}$
September	52	$1.45 \times 10^{-1} \pm 2.08 \times 10^{-1}$	$3.94 \times 10^{-4} \pm 3.81 \times 10^{-4}$	$9.11 \times 10^{-4} \pm 3.77 \times 10^{-4}$	$4.77 \times 10^{-2} \pm 4.51 \times 10^{-2}$	$1.77 \times 10^{-1} \pm 6.49 \times 10^{-2}$	$3.43 \times 10^{-2} \pm 1.74 \times 10^{-2}$
October	48	$3.81 \times 10^{-2} \pm 1.45 \times 10^{-1}$	$3.71 \times 10^{-4} \pm 2.10 \times 10^{-4}$	$1.59 \times 10^{-3} \pm 1.01 \times 10^{-3}$	$3.11 \times 10^{-2} \pm 3.47 \times 10^{-2}$	$2.04 \times 10^{-1} \pm 1.35 \times 10^{-1}$	$2.82 \times 10^{-2} \pm 1.61 \times 10^{-2}$
November	57	$1.26 \times 10^{-2} \pm 9.09 \times 10^{-3}$	$2.91 \times 10^{-4} \pm 2.26 \times 10^{-4}$	$2.24 \times 10^{-3} \pm 1.35 \times 10^{-3}$	$3.14 \times 10^{-2} \pm 1.63 \times 10^{-2}$	$2.48 \times 10^{-1} \pm 1.73 \times 10^{-1}$	$2.55 \times 10^{-2} \pm 1.34 \times 10^{-2}$
December	52	$1.25 \times 10^{-2} \pm 7.78 \times 10^{-3}$	$3.46 \times 10^{-4} \pm 2.91 \times 10^{-4}$	$1.94 \times 10^{-3} \pm 1.02 \times 10^{-3}$	$2.56 \times 10^{-2} \pm 1.02 \times 10^{-2}$	$2.39 \times 10^{-1} \pm 1.93 \times 10^{-1}$	$2.53 \times 10^{-2} \pm 1.37 \times 10^{-2}$

Table S1. Continued.

Month	n	EC3	OC1	OC2	OC3	OC4
January	58	$1.07 \times 10^{-3} \pm 7.57 \times 10^{-4}$	$1.17 \times 10^{-2} \pm 8.83 \times 10^{-3}$	$1.39 \times 10^{-1} \pm 8.81 \times 10^{-2}$	$2.41 \times 10^{-1} \pm 2.26 \times 10^{-1}$	$1.94 \times 10^{-1} \pm 1.74 \times 10^{-1}$
February	54	$2.00 \times 10^{-4} \pm 0.00 \times 10^{0}$	$2.65 \times 10^{-2} \pm 5.95 \times 10^{-2}$	$1.57 \times 10^{-1} \pm 1.10 \times 10^{-1}$	$2.48 \times 10^{-1} \pm 1.55 \times 10^{-1}$	$1.98 \times 10^{-1} \pm 1.18 \times 10^{-1}$
March	59	NaN	$8.57 \times 10^{-2} \pm 2.52 \times 10^{-1}$	$2.17 \times 10^{-1} \pm 3.43 \times 10^{-1}$	$3.05 \times 10^{-1} \pm 3.74 \times 10^{-1}$	$2.53 \times 10^{-1} \pm 2.51 \times 10^{-1}$
April	52	NaN	$2.71 \times 10^{-1} \pm 6.40 \times 10^{-1}$	$5.32 \times 10^{-1} \pm 1.36 \times 10^{0}$	$5.19 \times 10^{-1} \pm 8.41 \times 10^{-1}$	$3.35 \times 10^{-1} \pm 4.19 \times 10^{-1}$
May	52	$1.90 \times 10^{-3} \pm 2.58 \times 10^{-3}$	$2.98 \times 10^{-1} \pm 5.24 \times 10^{-1}$	$3.13 \times 10^{-1} \pm 7.91 \times 10^{-1}$	$3.43 \times 10^{-1} \pm 6.13 \times 10^{-1}$	$2.30 \times 10^{-1} \pm 2.71 \times 10^{-1}$
June	53	$3.00 \times 10^{-4} \pm 0.00 \times 10^{0}$	$6.51 \times 10^{-2} \pm 1.37 \times 10^{-1}$	$1.40 \times 10^{-1} \pm 1.61 \times 10^{-1}$	$2.19 \times 10^{-1} \pm 1.86 \times 10^{-1}$	$1.55 \times 10^{-1} \pm 1.18 \times 10^{-1}$
July	60	$1.10 \times 10^{-3} \pm 9.90 \times 10^{-4}$	$1.34 \times 10^{-2} \pm 1.27 \times 10^{-2}$	$1.09 \times 10^{-1} \pm 5.96 \times 10^{-2}$	$1.65 \times 10^{-1} \pm 1.05 \times 10^{-1}$	$1.26 \times 10^{-1} \pm 8.16 \times 10^{-2}$
August	61	NaN	$2.97 \times 10^{-2} \pm 3.16 \times 10^{-2}$	$9.80 \times 10^{-2} \pm 3.55 \times 10^{-2}$	$1.38 \times 10^{-1} \pm 6.30 \times 10^{-2}$	$1.08 \times 10^{-1} \pm 3.84 \times 10^{-2}$
September	52	$1.48 \times 10^{-3} \pm 1.32 \times 10^{-3}$	$1.25 \times 10^{-2} \pm 5.86 \times 10^{-3}$	$1.06 \times 10^{-1} \pm 2.90 \times 10^{-2}$	$1.60 \times 10^{-1} \pm 6.67 \times 10^{-2}$	$1.25 \times 10^{-1} \pm 3.39 \times 10^{-2}$
October	48	$3.00 \times 10^{-4} \pm 0.00 \times 10^{0}$	$2.07 \times 10^{-2} \pm 1.69 \times 10^{-2}$	$1.31 \times 10^{-1} \pm 6.58 \times 10^{-2}$	$1.90 \times 10^{-1} \pm 1.30 \times 10^{-1}$	$1.37 \times 10^{-1} \pm 7.07 \times 10^{-2}$
November	57	$4.70 \times 10^{-4} \pm 1.73 \times 10^{-5}$	$3.19 \times 10^{-2} \pm 4.38 \times 10^{-2}$	$1.69 \times 10^{-1} \pm 1.37 \times 10^{-1}$	$2.93 \times 10^{-1} \pm 2.75 \times 10^{-1}$	$2.04 \times 10^{-1} \pm 1.84 \times 10^{-1}$
December	52	$1.10 \times 10^{-3} \pm 8.46 \times 10^{-4}$	$2.83 \times 10^{-2} \pm 2.62 \times 10^{-2}$	$1.37 \times 10^{-1} \pm 7.74 \times 10^{-2}$	$2.17 \times 10^{-1} \pm 1.57 \times 10^{-1}$	$1.71 \times 10^{-1} \pm 1.21 \times 10^{-1}$

Table S1. Continued.

Month	n	Cl^-	Cr	Cu	Fe	Pb	Mg
January	58	$2.20 \times 10^{-1} \pm 3.25 \times 10^{-1}$	$9.18 \times 10^{-5} \pm 6.11 \times 10^{-5}$	$4.22 \times 10^{-4} \pm 3.01 \times 10^{-4}$	$8.17 \times 10^{-3} \pm 7.18 \times 10^{-3}$	$7.18 \times 10^{-4} \pm 5.26 \times 10^{-4}$	$4.53 \times 10^{-2} \pm 3.25 \times 10^{-2}$
February	54	$2.08 \times 10^{-1} \pm 2.89 \times 10^{-1}$	$8.47 \times 10^{-5} \pm 8.78 \times 10^{-5}$	$3.72 \times 10^{-4} \pm 2.46 \times 10^{-4}$	$9.45 \times 10^{-3} \pm 8.58 \times 10^{-3}$	$7.86 \times 10^{-4} \pm 5.63 \times 10^{-4}$	$5.03 \times 10^{-2} \pm 3.24 \times 10^{-2}$
March	59	$2.14 \times 10^{-1} \pm 3.12 \times 10^{-1}$	$1.70 \times 10^{-4} \pm 1.40 \times 10^{-4}$	$4.31 \times 10^{-4} \pm 3.68 \times 10^{-4}$	$1.81 \times 10^{-2} \pm 1.86 \times 10^{-2}$	$8.60 \times 10^{-4} \pm 5.59 \times 10^{-4}$	$4.85 \times 10^{-2} \pm 3.17 \times 10^{-2}$
April	52	$1.85 \times 10^{-1} \pm 2.42 \times 10^{-1}$	$1.91 \times 10^{-4} \pm 4.43 \times 10^{-4}$	$3.05 \times 10^{-4} \pm 2.03 \times 10^{-4}$	$3.47 \times 10^{-2} \pm 4.89 \times 10^{-2}$	$7.13 \times 10^{-4} \pm 6.07 \times 10^{-4}$	$4.89 \times 10^{-2} \pm 2.55 \times 10^{-2}$
May	52	$1.65 \times 10^{-1} \pm 2.46 \times 10^{-1}$	$2.65 \times 10^{-4} \pm 5.37 \times 10^{-4}$	$3.95 \times 10^{-4} \pm 6.84 \times 10^{-4}$	$6.15 \times 10^{-2} \pm 1.27 \times 10^{-1}$	$1.16 \times 10^{-3} \pm 3.81 \times 10^{-3}$	$5.31 \times 10^{-2} \pm 2.88 \times 10^{-2}$
June	53	$1.74 \times 10^{-1} \pm 2.38 \times 10^{-1}$	$4.32 \times 10^{-4} \pm 1.00 \times 10^{-3}$	$3.74 \times 10^{-4} \pm 3.71 \times 10^{-4}$	$1.37 \times 10^{-1} \pm 1.57 \times 10^{-1}$	$6.16 \times 10^{-4} \pm 4.67 \times 10^{-4}$	$4.97 \times 10^{-2} \pm 2.65 \times 10^{-2}$
July	60	$1.53 \times 10^{-1} \pm 1.76 \times 10^{-1}$	$4.29 \times 10^{-4} \pm 4.45 \times 10^{-4}$	$7.73 \times 10^{-4} \pm 2.02 \times 10^{-3}$	$1.85 \times 10^{-1} \pm 1.58 \times 10^{-1}$	$7.35 \times 10^{-4} \pm 5.66 \times 10^{-4}$	$5.30 \times 10^{-2} \pm 3.51 \times 10^{-2}$
August	61	$1.61 \times 10^{-1} \pm 1.74 \times 10^{-1}$	$2.84 \times 10^{-4} \pm 3.06 \times 10^{-4}$	$2.56 \times 10^{-4} \pm 2.18 \times 10^{-4}$	$1.39 \times 10^{-1} \pm 1.74 \times 10^{-1}$	$4.26 \times 10^{-4} \pm 2.47 \times 10^{-4}$	$5.28 \times 10^{-2} \pm 3.87 \times 10^{-2}$
September	52	$7.05 \times 10^{-2} \pm 1.10 \times 10^{-1}$	$1.90 \times 10^{-4} \pm 1.97 \times 10^{-4}$	$3.53 \times 10^{-4} \pm 2.74 \times 10^{-4}$	$8.14 \times 10^{-2} \pm 1.12 \times 10^{-1}$	$5.36 \times 10^{-4} \pm 3.94 \times 10^{-4}$	$3.42 \times 10^{-2} \pm 2.11 \times 10^{-2}$
October	48	$1.86 \times 10^{-1} \pm 2.74 \times 10^{-1}$	$1.22 \times 10^{-4} \pm 1.36 \times 10^{-4}$	$3.85 \times 10^{-4} \pm 2.47 \times 10^{-4}$	$2.37 \times 10^{-3} \pm 8.34 \times 10^{-2}$	$6.36 \times 10^{-4} \pm 4.64 \times 10^{-4}$	$4.03 \times 10^{-2} \pm 2.87 \times 10^{-2}$
November	57	$2.17 \times 10^{-1} \pm 3.17 \times 10^{-1}$	$1.11 \times 10^{-4} \pm 8.00 \times 10^{-5}$	$5.47 \times 10^{-4} \pm 3.67 \times 10^{-4}$	$9.18 \times 10^{-3} \pm 6.37 \times 10^{-3}$	$8.52 \times 10^{-4} \pm 7.01 \times 10^{-4}$	$4.71 \times 10^{-2} \pm 2.82 \times 10^{-2}$
December	52	$2.16 \times 10^{-1} \pm 3.13 \times 10^{-1}$	$9.12 \times 10^{-5} \pm 8.30 \times 10^{-5}$	$4.40 \times 10^{-4} \pm 3.54 \times 10^{-4}$	$7.97 \times 10^{-3} \pm 5.09 \times 10^{-3}$	$5.91 \times 10^{-4} \pm 4.15 \times 10^{-4}$	$4.55 \times 10^{-2} \pm 3.04 \times 10^{-2}$

Table S1. Continued.

Month	n	Mn	$PM_{2.5}$	Ni	NO_3^-	P	K
January	58	$2.85 \times 10^{-4} \pm 2.61 \times 10^{-4}$	4.33± 1.89	$3.33 \times 10^{-4} \pm 2.26 \times 10^{-4}$	$3.37 \times 10^{-1} \pm 1.39 \times 10^{-1}$	$6.55 \times 10^{-4} \pm 4.01 \times 10^{-4}$	$6.08 \times 10^{-2} \pm 6.31 \times 10^{-2}$
February	54	$3.82 \times 10^{-4} \pm 2.53 \times 10^{-4}$	4.83 ± 1.82	$4.55 \times 10^{-4} \pm 3.72 \times 10^{-4}$	$3.92 \times 10^{-1} \pm 1.71 \times 10^{-1}$	$9.14 \times 10^{-4} \pm 7.40 \times 10^{-4}$	$4.84 \times 10^{-2} \pm 2.58 \times 10^{-2}$
March	59	$5.54 \times 10^{-4} \pm 6.09 \times 10^{-4}$	6.20 ± 3.02	$3.79 \times 10^{-4} \pm 2.74 \times 10^{-4}$	$3.90 \times 10^{-1} \pm 1.64 \times 10^{-1}$	$2.45 \times 10^{-3} \pm 2.28 \times 10^{-3}$	$5.37 \times 10^{-2} \pm 2.97 \times 10^{-2}$
April	52	$8.46 \times 10^{-4} \pm 8.93 \times 10^{-4}$	7.12 ± 5.23	$5.04 \times 10^{-4} \pm 3.48 \times 10^{-4}$	$3.86 \times 10^{-1} \pm 1.39 \times 10^{-1}$	$1.56 \times 10^{-3} \pm 1.66 \times 10^{-3}$	$5.41 \times 10^{-2} \pm 3.41 \times 10^{-2}$
May	52	$1.19 \times 10^{-3} \pm 2.10 \times 10^{-3}$	6.74 ± 4.17	$4.49 \times 10^{-4} \pm 3.28 \times 10^{-4}$	$3.52 \times 10^{-1} \pm 1.46 \times 10^{-1}$	$1.98 \times 10^{-3} \pm 2.18 \times 10^{-3}$	$5.67 \times 10^{-2} \pm 7.37 \times 10^{-2}$
June	53	$2.63 \times 10^{-3} \pm 2.78 \times 10^{-3}$	6.68 ± 3.16	$5.69 \times 10^{-4} \pm 4.54 \times 10^{-4}$	$3.16 \times 10^{-1} \pm 1.18 \times 10^{-1}$	$1.15 \times 10^{-3} \pm 5.71 \times 10^{-4}$	$6.39 \times 10^{-2} \pm 5.42 \times 10^{-2}$
July	60	$3.02 \times 10^{-3} \pm 2.64 \times 10^{-3}$	7.30 ± 3.84	$4.85 \times 10^{-4} \pm 3.37 \times 10^{-4}$	$2.88 \times 10^{-1} \pm 1.07 \times 10^{-1}$	$7.74 \times 10^{-4} \pm 1.07 \times 10^{-3}$	$8.25 \times 10^{-2} \pm 8.49 \times 10^{-2}$
August	61	$2.36 \times 10^{-3} \pm 2.84 \times 10^{-3}$	5.90 ± 3.47	$4.21 \times 10^{-4} \pm 2.77 \times 10^{-4}$	$2.39 \times 10^{-1} \pm 8.81 \times 10^{-2}$	$1.09 \times 10^{-3} \pm 8.69 \times 10^{-4}$	$5.87 \times 10^{-2} \pm 5.02 \times 10^{-2}$
September	52	$1.42 \times 10^{-3} \pm 1.75 \times 10^{-3}$	4.48 ± 2.18	$4.21 \times 10^{-4} \pm 2.26 \times 10^{-4}$	$2.28 \times 10^{-1} \pm 8.25 \times 10^{-2}$	$1.02 \times 10^{-3} \pm 6.71 \times 10^{-4}$	$4.41 \times 10^{-2} \pm 3.93 \times 10^{-2}$
October	48	$6.85 \times 10^{-4} \pm 1.68 \times 10^{-3}$	4.40 ± 2.13	$3.69 \times 10^{-4} \pm 2.69 \times 10^{-4}$	$2.47 \times 10^{-1} \pm 9.50 \times 10^{-2}$	$6.71 \times 10^{-4} \pm 6.36 \times 10^{-4}$	$3.59 \times 10^{-2} \pm 3.68 \times 10^{-2}$
November	57	$3.27 \times 10^{-4} \pm 2.57 \times 10^{-4}$	4.47 ± 1.87	$3.49 \times 10^{-4} \pm 2.17 \times 10^{-4}$	$3.26 \times 10^{-1} \pm 1.15 \times 10^{-1}$	$5.70 \times 10^{-4} \pm 4.10 \times 10^{-4}$	$5.07 \times 10^{-2} \pm 4.05 \times 10^{-2}$
December	52	$3.15 \times 10^{-4} \pm 2.16 \times 10^{-4}$	4.14 ± 1.30	$3.74 \times 10^{-4} \pm 2.28 \times 10^{-4}$	$2.96 \times 10^{-1} \pm 9.76 \times 10^{-2}$	$6.01 \times 10^{-4} \pm 5.20 \times 10^{-4}$	$5.12 \times 10^{-2} \pm 4.44 \times 10^{-2}$

Table S1. Continued.

Month	n	Rb	Se	Si	Na	Sr
January	58	$1.32 \times 10^{-4} \pm 1.03 \times 10^{-4}$	$1.86 \times 10^{-4} \pm 1.21 \times 10^{-4}$	$1.89 \times 10^{-2} \pm 3.06 \times 10^{-2}$	$2.89 \times 10^{-1} \pm 2.03 \times 10^{-1}$	$4.54 \times 10^{-4} \pm 2.91 \times 10^{-4}$
February	54	$2.09 \times 10^{-4} \pm 1.23 \times 10^{-4}$	$2.28 \times 10^{-4} \pm 1.28 \times 10^{-4}$	$1.96 \times 10^{-2} \pm 3.90 \times 10^{-2}$	$3.18 \times 10^{-1} \pm 2.07 \times 10^{-1}$	$4.45 \times 10^{-4} \pm 4.14 \times 10^{-4}$
March	59	$1.99 \times 10^{-4} \pm 1.63 \times 10^{-4}$	$2.67 \times 10^{-4} \pm 1.41 \times 10^{-4}$	$4.17 \times 10^{-2} \pm 4.35 \times 10^{-2}$	$3.09 \times 10^{-1} \pm 2.03 \times 10^{-1}$	$1.26 \times 10^{-3} \pm 2.46 \times 10^{-3}$
April	52	$1.65 \times 10^{-4} \pm 1.23 \times 10^{-4}$	$2.22 \times 10^{-4} \pm 1.35 \times 10^{-4}$	$1.05 \times 10^{-1} \pm 1.76 \times 10^{-1}$	$3.22 \times 10^{-1} \pm 1.72 \times 10^{-1}$	$7.43 \times 10^{-4} \pm 4.60 \times 10^{-4}$
May	52	$2.57 \times 10^{-4} \pm 2.13 \times 10^{-4}$	$2.45 \times 10^{-4} \pm 1.42 \times 10^{-4}$	$1.90 \times 10^{-1} \pm 3.69 \times 10^{-1}$	$2.96 \times 10^{-1} \pm 1.60 \times 10^{-1}$	$7.37 \times 10^{-4} \pm 9.15 \times 10^{-4}$
June	53	$3.51 \times 10^{-4} \pm 2.56 \times 10^{-4}$	$1.83 \times 10^{-4} \pm 1.07 \times 10^{-4}$	$4.46 \times 10^{-1} \pm 5.11 \times 10^{-1}$	$2.61 \times 10^{-1} \pm 1.46 \times 10^{-1}$	$1.04 \times 10^{-3} \pm 9.56 \times 10^{-3}$
July	60	$3.13 \times 10^{-4} \pm 2.24 \times 10^{-4}$	$2.06 \times 10^{-4} \pm 1.55 \times 10^{-4}$	$6.11 \times 10^{-1} \pm 5.09 \times 10^{-1}$	$2.34 \times 10^{-1} \pm 1.20 \times 10^{-1}$	$1.72 \times 10^{-3} \pm 2.04 \times 10^{-3}$
August	61	$3.03 \times 10^{-4} \pm 2.99 \times 10^{-4}$	$1.74 \times 10^{-4} \pm 1.14 \times 10^{-4}$	$4.53 \times 10^{-1} \pm 5.59 \times 10^{-1}$	$2.19 \times 10^{-1} \pm 1.19 \times 10^{-1}$	$1.22 \times 10^{-3} \pm 1.23 \times 10^{-3}$
September	52	$2.11 \times 10^{-4} \pm 1.74 \times 10^{-4}$	$1.70 \times 10^{-4} \pm 1.24 \times 10^{-4}$	$2.84 \times 10^{-1} \pm 3.98 \times 10^{-1}$	$1.60 \times 10^{-1} \pm 9.09 \times 10^{-2}$	$7.67 \times 10^{-4} \pm 7.21 \times 10^{-4}$
October	48	$1.61 \times 10^{-4} \pm 1.56 \times 10^{-4}$	$1.83 \times 10^{-4} \pm 1.19 \times 10^{-4}$	$7.13 \times 10^{-2} \pm 3.11 \times 10^{-1}$	$2.59 \times 10^{-1} \pm 1.93 \times 10^{-1}$	$4.60 \times 10^{-4} \pm 5.73 \times 10^{-4}$
November	57	$1.47 \times 10^{-4} \pm 9.21 \times 10^{-5}$	$2.04 \times 10^{-4} \pm 1.26 \times 10^{-4}$	$1.34 \times 10^{-2} \pm 1.45 \times 10^{-2}$	$2.90 \times 10^{-1} \pm 1.81 \times 10^{-1}$	$4.57 \times 10^{-4} \pm 2.54 \times 10^{-4}$
December	52	$1.23 \times 10^{-4} \pm 1.02 \times 10^{-4}$	$2.05 \times 10^{-4} \pm 1.32 \times 10^{-4}$	$1.14 \times 10^{-2} \pm 9.78 \times 10^{-3}$	$2.89 \times 10^{-1} \pm 1.91 \times 10^{-1}$	$3.83 \times 10^{-4} \pm 2.43 \times 10^{-4}$

Table S1. Continued.

Month	n	SO_4^{2-}	Ti	V	Zn	Zr
January	58	$1.08 \pm 6.11 \times 10^{-1}$	$7.19 \times 10^{-4} \pm 6.85 \times 10^{-4}$	$1.17 \times 10^{-3} \pm 9.23 \times 10^{-4}$	$1.88 \times 10^{-3} \pm 1.56 \times 10^{-3}$	$7.85 \times 10^{-4} \pm 5.79 \times 10^{-4}$
February	54	$1.37 \pm 8.60 \times 10^{-1}$	$8.02 \times 10^{-4} \pm 1.03 \times 10^{-3}$	$1.74 \times 10^{-3} \pm 1.50 \times 10^{-3}$	$1.98 \times 10^{-3} \pm 1.66 \times 10^{-3}$	$7.39 \times 10^{-4} \pm 4.53 \times 10^{-4}$
March	59	$1.61 \pm 8.14 \times 10^{-1}$	$1.77 \times 10^{-3} \pm 2.00 \times 10^{-3}$	$1.27 \times 10^{-3} \pm 1.03 \times 10^{-3}$	$2.07 \times 10^{-3} \pm 1.44 \times 10^{-3}$	$6.84 \times 10^{-4} \pm 4.89 \times 10^{-4}$
April	52	$1.64 \pm 9.61 \times 10^{-1}$	$3.75 \times 10^{-3} \pm 6.07 \times 10^{-3}$	$1.68 \times 10^{-3} \pm 1.20 \times 10^{-3}$	$1.65 \times 10^{-3} \pm 1.18 \times 10^{-3}$	$6.76 \times 10^{-4} \pm 5.50 \times 10^{-4}$
May	52	$1.53 \pm 7.61 \times 10^{-1}$	$6.72 \times 10^{-3} \pm 1.41 \times 10^{-2}$	$1.59 \times 10^{-3} \pm 1.06 \times 10^{-3}$	$1.90 \times 10^{-3} \pm 3.23 \times 10^{-3}$	$7.13 \times 10^{-4} \pm 5.40 \times 10^{-4}$
June	53	$1.20 \pm 4.46 \times 10^{-1}$	$1.64 \times 10^{-2} \pm 1.90 \times 10^{-2}$	$1.92 \times 10^{-3} \pm 9.60 \times 10^{-4}$	$1.62 \times 10^{-3} \pm 2.75 \times 10^{-3}$	$9.41 \times 10^{-4} \pm 7.47 \times 10^{-4}$
July	60	$1.28 \pm 8.96 \times 10^{-1}$	$2.12 \times 10^{-2} \pm 1.85 \times 10^{-2}$	$1.69 \times 10^{-3} \pm 1.06 \times 10^{-3}$	$1.17 \times 10^{-3} \pm 1.04 \times 10^{-3}$	$1.21 \times 10^{-3} \pm 9.77 \times 10^{-4}$
August	61	$1.07 \pm 4.09 \times 10^{-1}$	$1.51 \times 10^{-2} \pm 1.90 \times 10^{-2}$	$1.49 \times 10^{-3} \pm 8.31 \times 10^{-4}$	$7.98 \times 10^{-4} \pm 6.20 \times 10^{-4}$	$8.82 \times 10^{-4} \pm 7.04 \times 10^{-4}$
September	52	$1.08 \pm 5.66 \times 10^{-1}$	$8.32 \times 10^{-3} \pm 1.16 \times 10^{-2}$	$1.56 \times 10^{-3} \pm 9.18 \times 10^{-4}$	$1.06 \times 10^{-3} \pm 5.98 \times 10^{-4}$	$7.14 \times 10^{-4} \pm 5.78 \times 10^{-4}$
October	48	$1.20 \pm 5.06 \times 10^{-1}$	$2.39 \times 10^{-3} \pm 9.67 \times 10^{-3}$	$1.26 \times 10^{-3} \pm 9.02 \times 10^{-4}$	$1.46 \times 10^{-3} \pm 1.06 \times 10^{-3}$	$5.71 \times 10^{-4} \pm 4.84 \times 10^{-4}$
November	57	$9.70 \times 10^{-1} \pm 3.39 \times 10^{-1}$	$7.37 \times 10^{-4} \pm 5.80 \times 10^{-4}$	$1.05 \times 10^{-3} \pm 7.31 \times 10^{-4}$	$1.97 \times 10^{-3} \pm 1.46 \times 10^{-3}$	$5.05 \times 10^{-4} \pm 4.55 \times 10^{-4}$
December	52	$9.68 \times 10^{-1} \pm 3.61 \times 10^{-1}$	$6.24 \times 10^{-4} \pm 4.16 \times 10^{-4}$	$1.26 \times 10^{-3} \pm 8.36 \times 10^{-4}$	$1.74 \times 10^{-3} \pm 1.47 \times 10^{-3}$	$6.79 \times 10^{-4} \pm 6.00 \times 10^{-4}$

Table S2. Monthly mean and standard deviation for the precipitation chemistry data (NADP). Number of data points available is shown as "n".

Month	n	pH	Ca^{2+}	Mg^{2+}	K^{+}	Na^{+}	NH_4^{+}	NO_3^{-}	Cl^{-}	SO_4^{2-}
January	15	5.36 ± 0.62	0.21 ± 0.31	0.13 ± 0.22	0.15 ± 0.14	1.01 ±1.87	0.33 ± 0.19	0.55 ± 0.56	1.84 ± 3.30	0.98 ± 0.69
February	11	5.32 ± 0.45	0.14 ± 0.28	0.11 ± 0.22	0.06 ± 0.12	0.94 ± 1.92	0.19 ± 0.18	0.56 ± 0.57	1.66 ± 3.31	1.10 ± 0.80
March	14	0.31 ± 0.31	0.19 ± 0.22	0.07 ± 0.22	0.04 ± 0.09	0.56 ± 1.89	0.30 ± 0.20	0.83 ± 0.69	1.00 ± 3.26	1.05 ± 1.08
April	16	5.35 ± 0.51	0.15 ± 0.17	0.08 ± 0.16	0.05 ± 0.06	0.60 ±1.32	0.26 ± 0.18	0.59 ± 0.28	1.10 ± 2.41	0.88 ± 0.44
May	22	5.58 ± 0.46	0.10 ± 0.17	0.08 ± 0.05	0.11 ± 0.08	0.64 ± 0.41	0.20 ± 0.24	0.40 ± 0.54	1.15 ± 0.77	0.54 ± 0.27
June	22	5.22 ± 0.45	0.13 ± 0.11	0.06 ± 0.11	0.07 ± 0.06	0.44 ± 0.91	0.19 ± 0.13	0.66 ± 0.48	0.79 ± 1.61	0.64 ± 0.38
July	25	5.14 ± 0.34	0.12 ± 0.20	0.06 ± 0.07	0.07 ± 0.16	0.46 ± 0.57	0.10 ± 0.08	0.64 ± 0.52	0.83 ± 1.01	0.56 ± 0.42
August	24	5.38 ± 0.60	0.12 ± 0.14	0.06 ± 0.08	0.18 ± 0.43	0.40 ± 0.53	0.15 ± 0.36	0.49 ± 0.30	0.74 ± 0.97	0.47 ± 0.32
September	20	5.34 ± 0.52	0.10 ± 0.07	0.06 ± 0.05	0.10 ± 0.22	0.39 ± 0.23	0.14 ± 0.46	0.51 ± 0.34	0.71 ± 0.39	0.56 ± 0.29
October	14	5.34 ± 0.59	0.15 ± 0.54	0.21 ± 0.32	0.09 ± 0.20	1.74 ± 2.80	0.14 ± 0.24	0.50 ± 1.59	3.07 ± 4.92	0.92 ± 1.12
November	15	5.39 ± 0.57	0.10 ± 0.20	0.11 ± 0.25	0.19 ± 0.23	0.83 ± 2.22	0.24 ± 0.27	0.50 ±0.54	1.46 ± 3.84	0.69 ± 1.47
December	15	5.39 ± 0.51	0.09 ± 0.11	0.08 ± 0.07	0.16 ± 0.20	0.61 ± 0.64	0.12 ± 0.34	0.28 ± 0.31	1.11 ± 1.11	0.40 ± 0.36

Table S3. Method detection limit (MDL) for rain species measured by NADP/NTN.

Analyte	MDL (mg L^{-1})
Ca^{2+}	0.009
Mg^{2+}	0.002
K^{+}	0.004
Na^{+}	0.003
NH_4^{+}	0.019
NO_3^{-}	0.005
Cl^{-}	0.005
SO_4^{2-}	0.004

Table S4. Method detection limit (MDL) for aerosol species measured by IMPROVE. Lack of data is shown as “NaN”.

Analyte	MDL (μg m^{-3})
Al	0.00567
As	0.0002
Br	0.00017
Ca	0.00321
EC1	0.0092
EC2	0.0092
EC3	0.0092
OC1	0.0313
OC2	0.0313
OC3	0.0369
OC4	0.0313
Cl^{-}	0.00028
Cr	0.00018
Cu	0.00022
Fe	0.00134
Pb	0.00067
Mg	0.00234
Mn	0.00033
$PM_{2.5}$	NaN
Ni	0.00011
NO_3^{-}	0.0111
P	0.00022
K	0.00114
Rb	0.00022
Se	0.00022
Si	0.00241
Na	0.00413
Sr	0.00022
SO_4^{2-}	0.0124
Ti	0.00033
V	0.00011
Zn	0.00022
Zr	0.00134

Table S5. Summary of PMF results for varying number of factors. Source factor profile results are shown in Figures S7 – S9 for solutions with 3, 4, and 5 factors. Figure 3 shows results for the 6 factor solution.

	**Number of Factors**				
**Diagnostic**	**3**	**4**	**5**	**6**	**7**
Q_{robust}	28,213.8	19,525.1	16,228.0	13,738.1	11,671.4
Q_{true}	29,092	19,946.5	16,494.7	14,003.2	11853.9
Q_{true}/Q_{exp}	2.23	1.61	1.41	1.27	1.15
$Q/Q_{exp} > 6$	0	0	0	0	0
DISP %dQ	-182.5×10^{-5}	-2.045×10^{-5}	0	0	-2.96×10^{-1}
DISP Swaps	0	0	0	0	3
Factors with BS Mapping <100%	1	0	1	3	7
Factors with BS Mapping <80%	0	0	1	2	4
BS-DISP in Best Fit	0	0	0	1	6

Table S6. Summary statistics for $PM_{2.5}$ and speciated mass concentrations (μg m^{-3}) included in the PMF analysis from the Everglades National Park IMPROVE station between 2013 and 2018.

Species	Category	Signal/Noise	Minimum	25th Percentile	Median	75th Percentile	Maximum
Al	Strong	7.46	-1.55×10^{-3}	1.12×10^{-2}	2.15×10^{-2}	7.85×10^{-2}	1.37×10^{0}
As	Weak	0.47	0.00×10^{0}	0.00×10^{0}	0.00×10^{0}	2.80×10^{-4}	3.03×10^{-3}
Br	Strong	7.06	1.50×10^{-4}	8.90×10^{-4}	1.48×10^{-3}	2.65×10^{-3}	1.57×10^{-2}
Ca	Strong	9.37	5.24×10^{-3}	2.20×10^{-2}	3.28×10^{-2}	5.13×10^{-2}	1.28×10^{0}
EC1	Strong	7.71	1.39×10^{-2}	9.78×10^{-2}	1.67×10^{-1}	2.90×10^{-1}	2.67×10^{0}
EC2	Strong	2.31	-6.22×10^{-3}	1.31×10^{-2}	2.53×10^{-2}	3.67×10^{-2}	1.64×10^{-1}
EC3	Bad	0.12	0.00×10^{0}	0.00×10^{0}	0.00×10^{0}	0.00×10^{0}	6.30×10^{-3}
OC1	Bad	0.21	-5.18×10^{-2}	-2.11×10^{-2}	-9.51×10^{-3}	7.80×10^{-3}	1.42×10^{0}
OC2	Strong	3.88	2.27×10^{-2}	8.36×10^{-2}	1.14×10^{-1}	1.70×10^{-1}	2.92×10^{0}
OC3	Strong	3.90	-8.04×10^{-3}	1.12×10^{-1}	1.75×10^{-1}	2.70×10^{-1}	8.79×10^{0}
OC4	Strong	4.21	2.02×10^{-2}	9.39×10^{-2}	1.35×10^{-1}	2.01×10^{-1}	4.49×10^{0}
Cl^{-}	Strong	5.35	1.10×10^{-4}	4.53×10^{-3}	5.99×10^{-2}	2.64×10^{-1}	2.11×10^{0}
Cr	Weak	0.76	-2.10×10^{-4}	1.00×10^{-5}	8.00×10^{-5}	1.80×10^{-4}	1.41×10^{0}
Cu	Strong	1.55	-1.70×10^{-4}	1.50×10^{-4}	2.80×10^{-4}	5.00×10^{-4}	1.95×10^{1}
Fe	Strong	7.71	6.50×10^{-4}	6.95×10^{-3}	1.55×10^{-2}	4.67×10^{-2}	2.17×10^{0}
Pb	Weak	0.63	-7.60×10^{-4}	1.30×10^{-4}	4.35×10^{-4}	8.33×10^{-4}	1.53×10^{0}
Mg	Strong	5.14	-1.92×10^{-3}	2.44×10^{-2}	4.14×10^{-2}	6.15×10^{-2}	6.77×10^{-3}
Mn	Strong	1.92	-4.30×10^{-4}	1.90×10^{-4}	4.30×10^{-4}	9.73×10^{-4}	1.47×10^{-2}
$PM_{2.5}$	Weak	9.96	1.41×10^{0}	3.60×10^{0}	4.67×10^{0}	6.37×10^{0}	7.94×10^{-1}
Ni	Strong	2.86	-1.00×10^{-4}	2.20×10^{-4}	3.60×10^{-4}	5.60×10^{-4}	2.60×10^{-2}
NO_3^{-}	Strong	9.77	7.55×10^{-2}	2.27×10^{-1}	2.89×10^{-1}	3.82×10^{-1}	1.76×10^{-1}
P	Weak	0.81	0.00×10^{0}	0.00×10^{0}	0.00×10^{0}	6.73×10^{-4}	1.32×10^{-2}
K	Strong	9.99	6.89×10^{-3}	2.69×10^{-2}	3.87×10^{-2}	6.54×10^{-2}	1.07×10^{2}
Rb	Bad	0.28	-3.20×10^{-4}	-1.00×10^{-4}	4.00×10^{-5}	2.00×10^{-4}	3.34×10^{1}
Se	Bad	0.44	-1.90×10^{-4}	6.00×10^{-5}	1.70×10^{-4}	2.70×10^{-4}	3.35×10^{-3}
Si	Strong	6.01	-2.73×10^{-3}	8.57×10^{-3}	3.12×10^{-2}	1.56×10^{-1}	1.21×10^{0}
Na	Strong	6.07	1.52×10^{-2}	1.35×10^{-1}	2.28×10^{-1}	3.63×10^{-1}	7.94×10^{-3}
Sr	Strong	2.25	-1.90×10^{-4}	2.90×10^{-4}	4.85×10^{-4}	8.20×10^{-4}	5.90×10^{-1}
SO_4^{2-}	Strong	9.88	2.29×10^{-1}	8.13×10^{-1}	1.08×10^{0}	1.49×10^{0}	1.19×10^{-3}
Ti	Strong	4.50	-4.00×10^{-5}	5.80×10^{-4}	1.31×10^{-3}	4.81×10^{-3}	6.70×10^{-4}
V	Strong	5.50	-3.00×10^{-5}	7.78×10^{-4}	1.29×10^{-3}	1.90×10^{-3}	2.34×10^{0}
Zn	Strong	5.05	-2.00×10^{-5}	6.20×10^{-4}	1.16×10^{-3}	2.09×10^{-3}	9.19×10^{-1}
Zr	Bad	0.15	-1.30×10^{-3}	-2.10×10^{-4}	2.45×10^{-4}	8.00×10^{-4}	1.41×10^{-2}

Table S7. Volume weighted concentrations (mg L^{-1}) of Cl^-, Na^+, and K^+, and the Cl^-:Na^+ ratio in wet deposition samples collected for the month of October (2013–2018). Aerosol concentrations of K (µg m^{-3}) and the PMF combustion factor are included in the grey shaded area. Rows are bolded for the five highest concentrations for Cl^- and Na^+.

Start Date	End Date	K^+	Na^+	Cl^-	Cl^-:Na^+	K	Combustion
10/22/2013 14:00	10/29/2013 14:15	0.10	0.87	1.67	1.91	0.01	NA
10/21/2014 13:45	10/28/2014 14:00	0.11	0.53	0.96	1.81	0.01	NA
10/6/2015 13:45	10/13/2015 13:45	0.03	0.44	0.64	1.46	0.03	NA
**10/13/2015 13:45**	**10/20/2015 13:45**	**0.13**	**2.86**	**5.62**	**1.97**	**0.04**	**NA**
**10/20/2015 13:45**	**10/27/2015 13:45**	**0.12**	**9.31**	**16.21**	**1.74**	**0.04**	**2.70**
10/27/2015 13:45	11/3/2015 14:15	0.79	0.61	1.08	1.78	0.04	0.98
10/4/2016 13:20	10/11/2016 13:30	0.05	0.56	1.00	1.77	0.03	0.50
**10/11/2016 13:30**	**10/18/2016 13:15**	**0.02**	**5.09**	**9.26**	**1.82**	**0.02**	**3.26**
**10/18/2016 13:15**	**10/25/2016 13:30**	**0.04**	**7.07**	**12.44**	**1.76**	**0.02**	**2.70**
**10/25/2016 13:30**	**11/1/2016 13:30**	**0.22**	**2.52**	**4.28**	**1.70**	**0.04**	**4.97**
10/31/2017 13:30	11/7/2017 15:30	0.35	1.29	2.09	1.62	0.02	2.85
10/2/2018 13:00	10/9/2018 13:30	0.10	1.17	2.19	1.87	0.03	3.32
10/9/2018 13:30	10/16/2018 13:00	0.05	0.84	1.55	1.84	0.02	NA
10/30/2018 13:15	11/6/2018 14:00	0.05	0.53	1.04	1.96	0.03	1.70

Note:
NA = no model output available

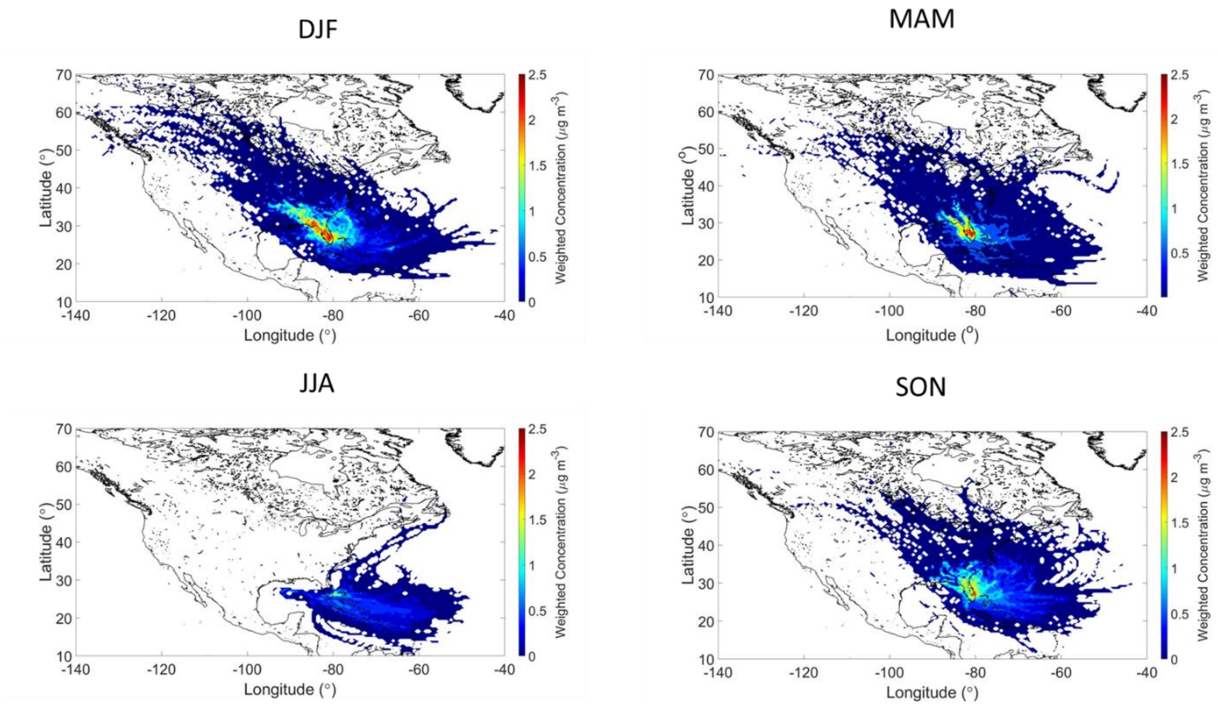

Figure S1. Combustion seasonal weight concentration weighted trajectory (WCWT) maps.

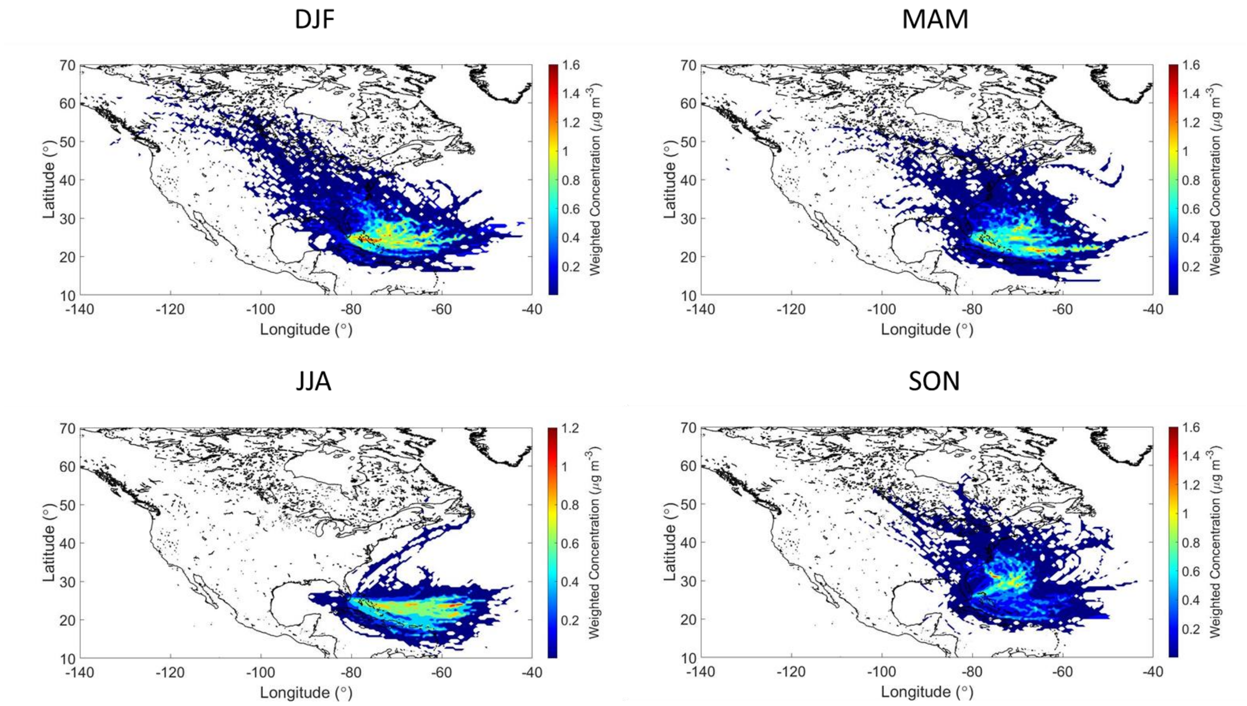

Figure S2. Same as Figure S1 for fresh sea salt.

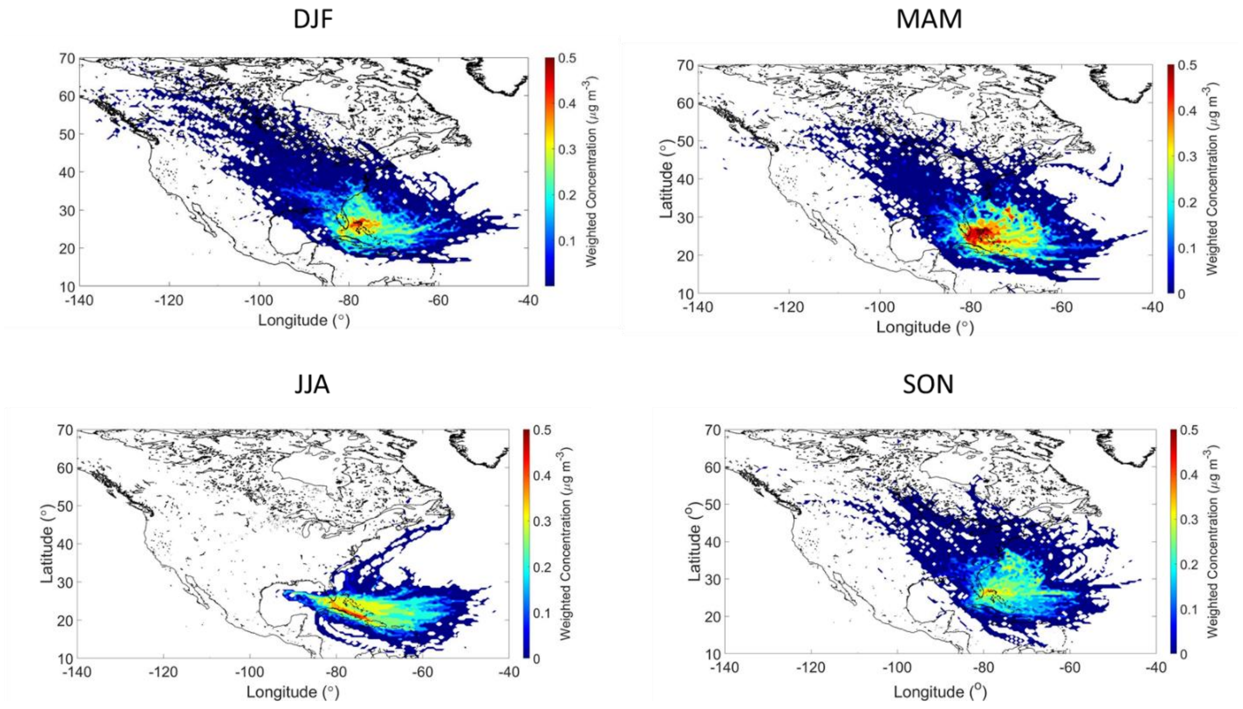

Figure S3. Same as Figure S1 for aged sea salt.

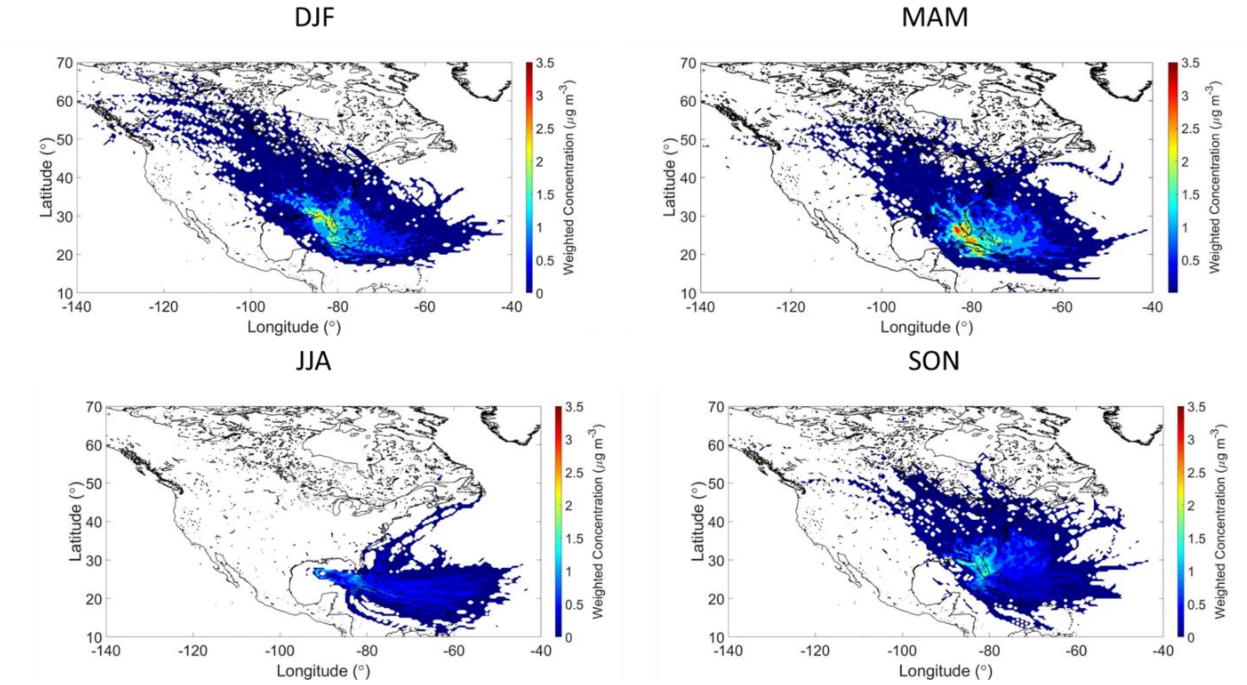

Figure S4. Same as Figure S1 for secondary sulfate.

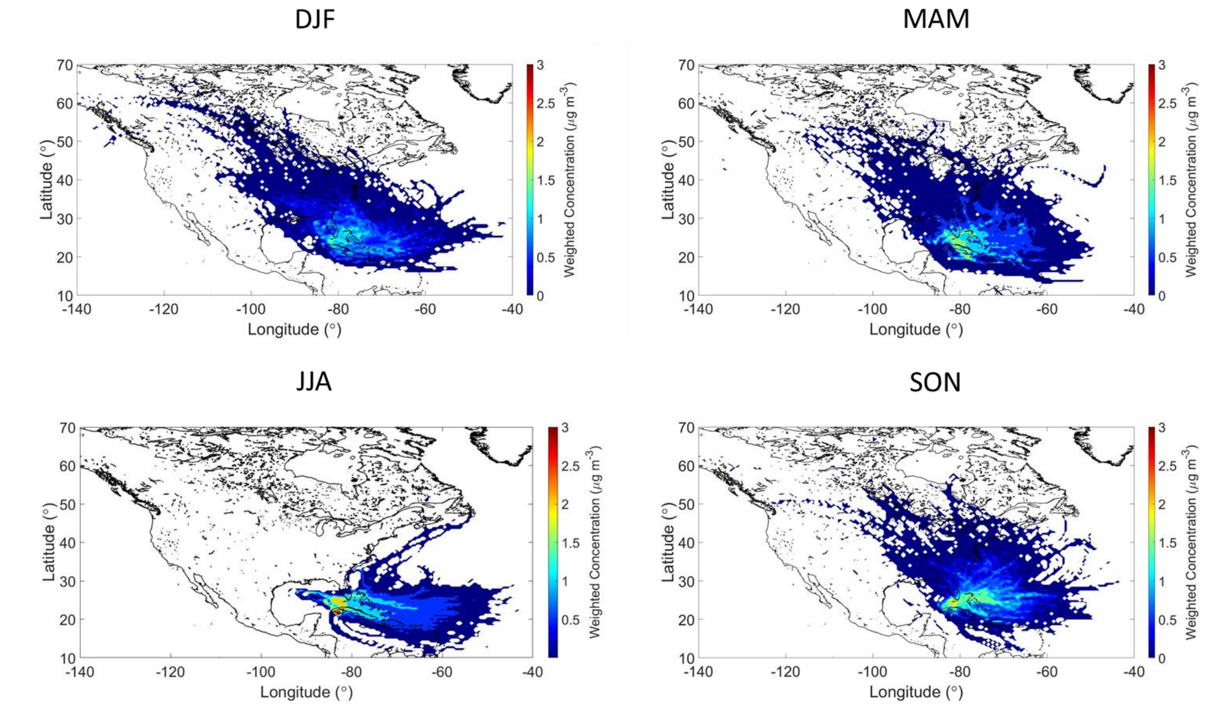

Figure S5. Same as Figure S1 for shipping emissions.

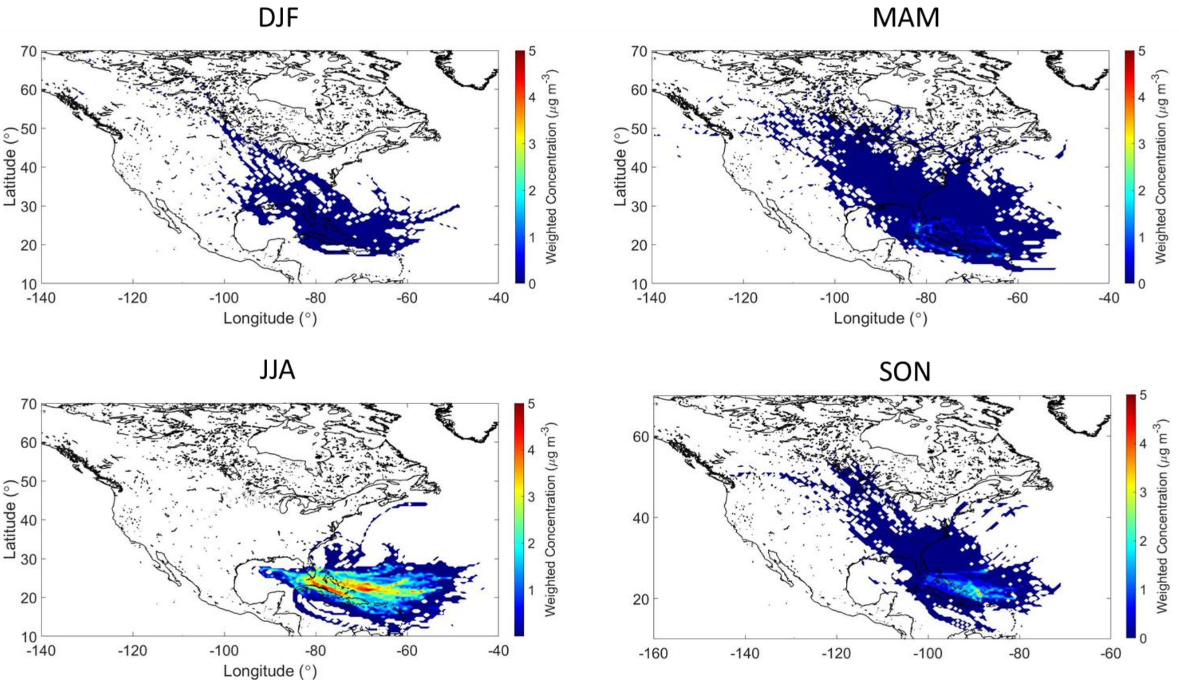

Figure S6. Same as Figure S1 for dust.

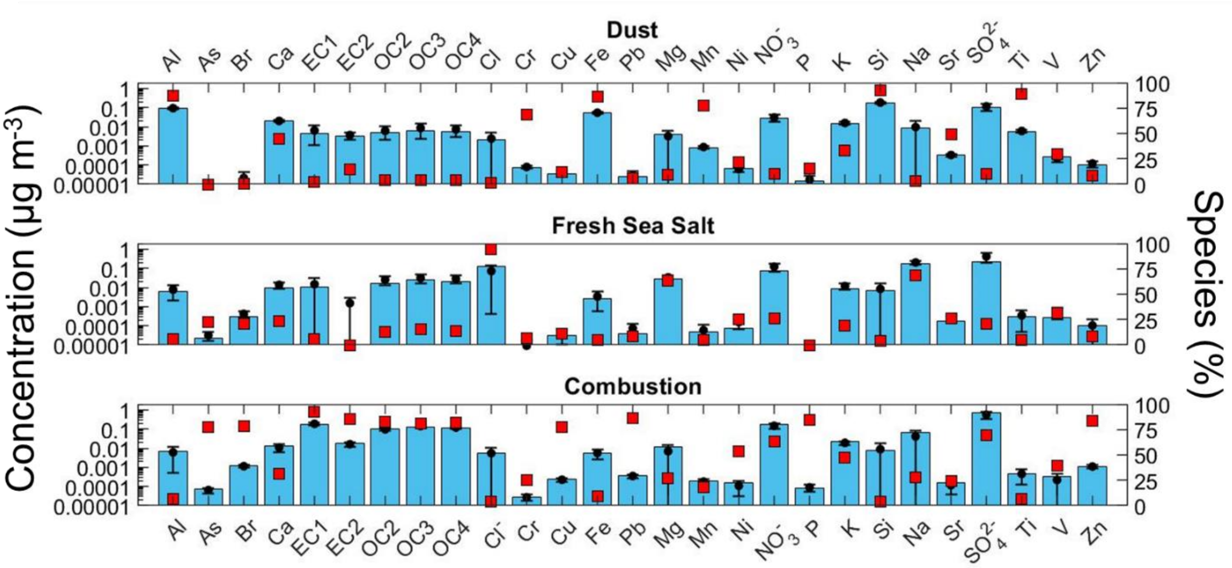

Figure S7. PMF analysis using EPA IMPROVE data from the Everglades NP station for 3 factors. Blue bars represent species concentrations; error bars show the maximum and minimum values and black markers represent the average DISP values. Red markers show the percent contribution from a particular source factor to each species' overall concentration.

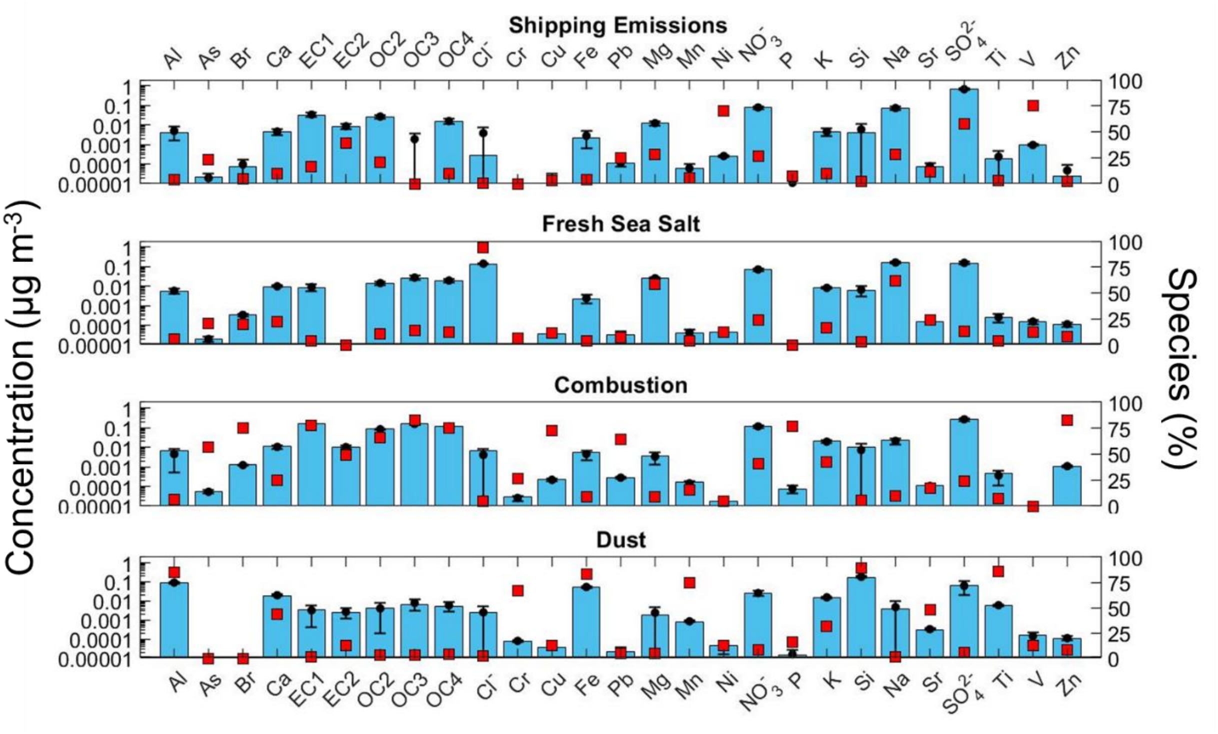

Figure S8. Same as Figure S7 but for 4 factors.

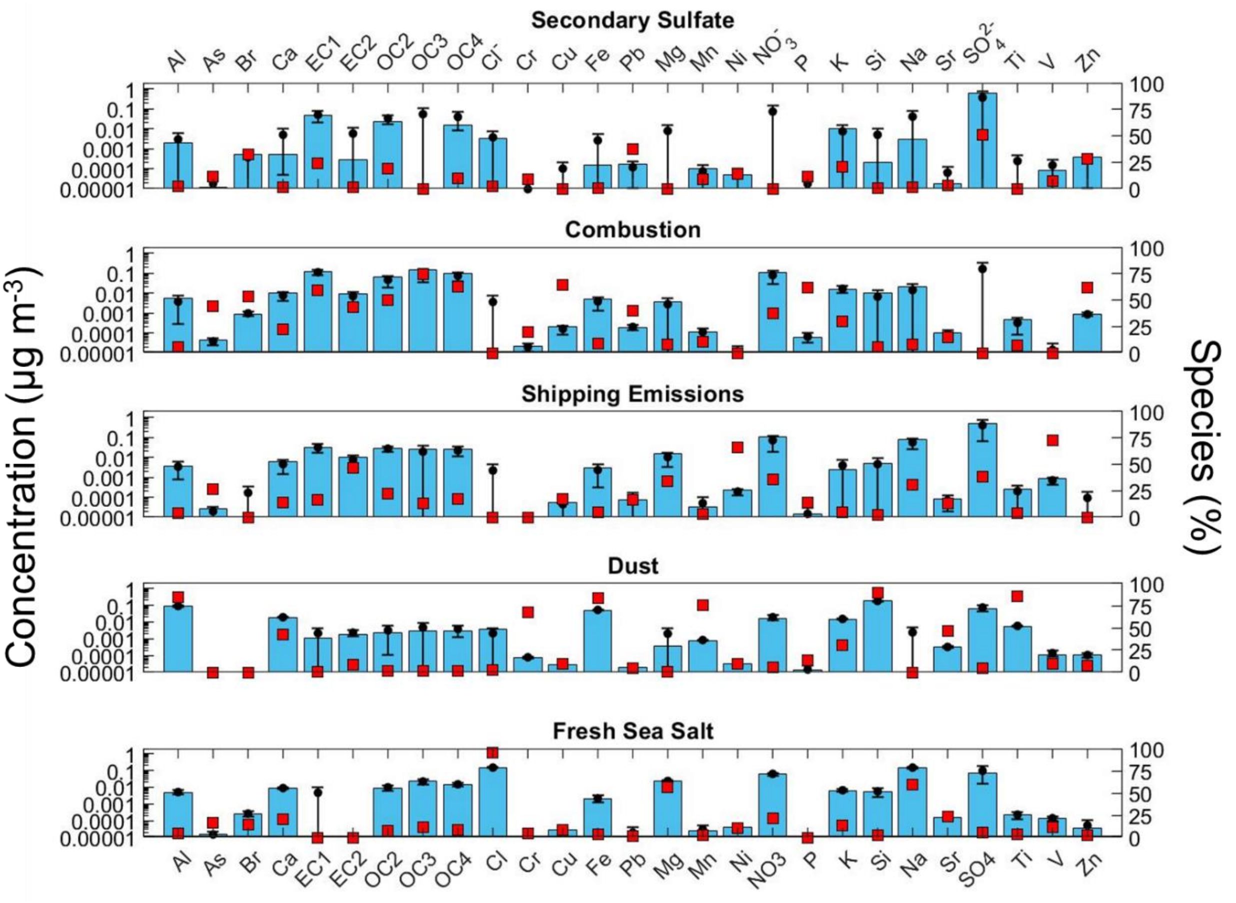

Figure S9. Same as Figure S7 but for 5 factors.

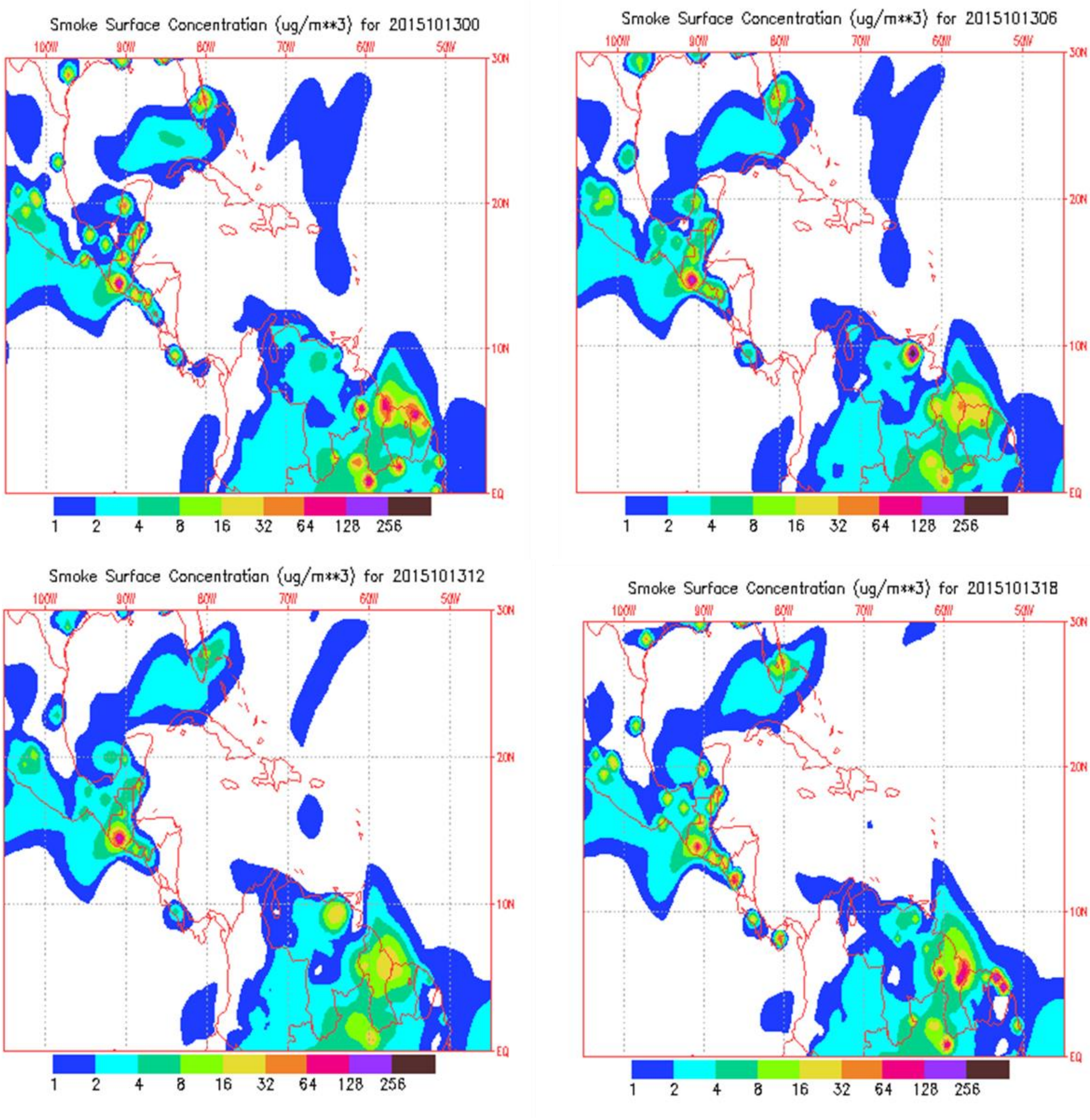

Figure S10. NAAPS smoke surface concentration (μg m^{-3}) for 13 October 2015.

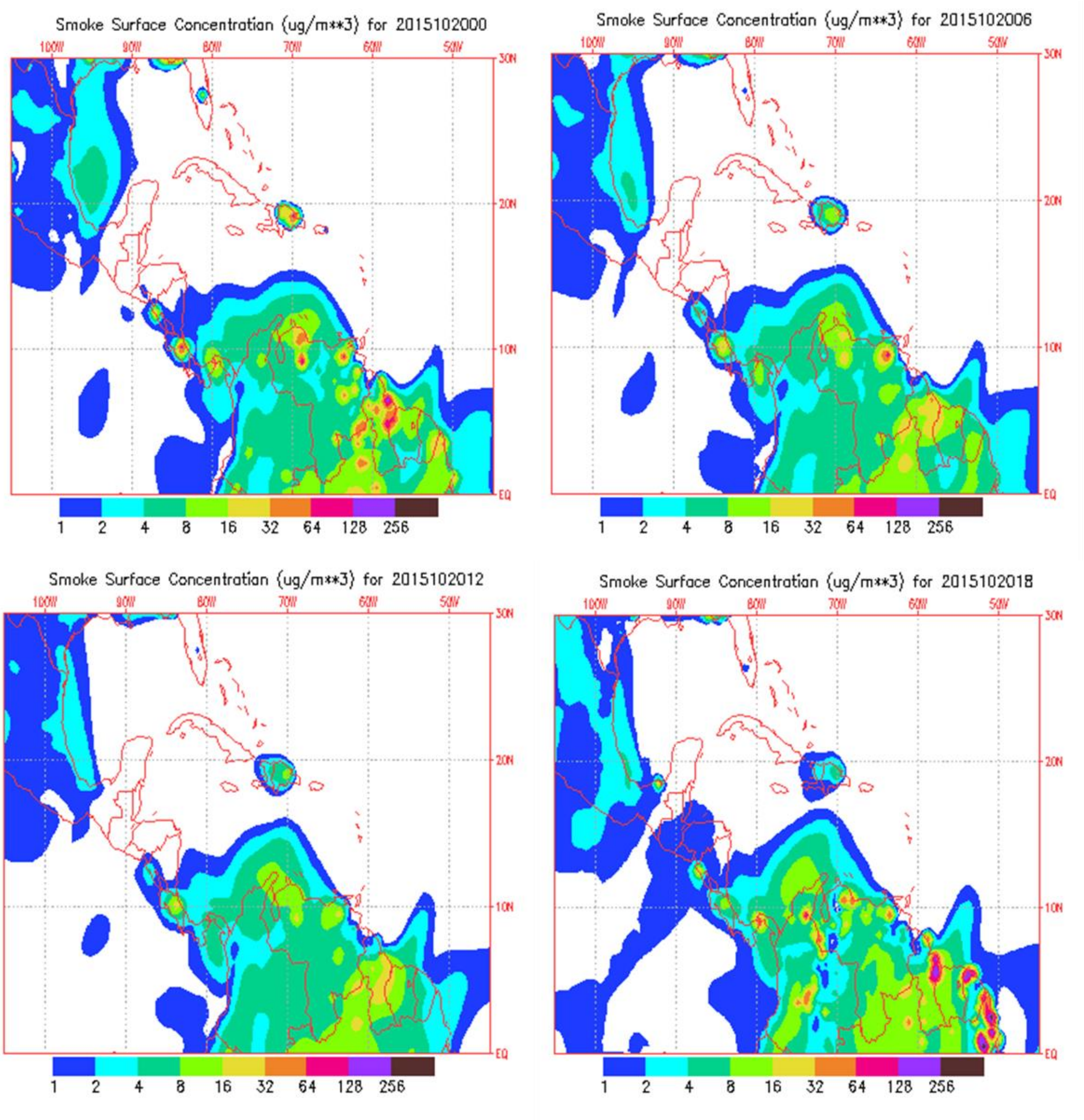

Figure S11. Same as Figure S10 but for 20 October 2015.

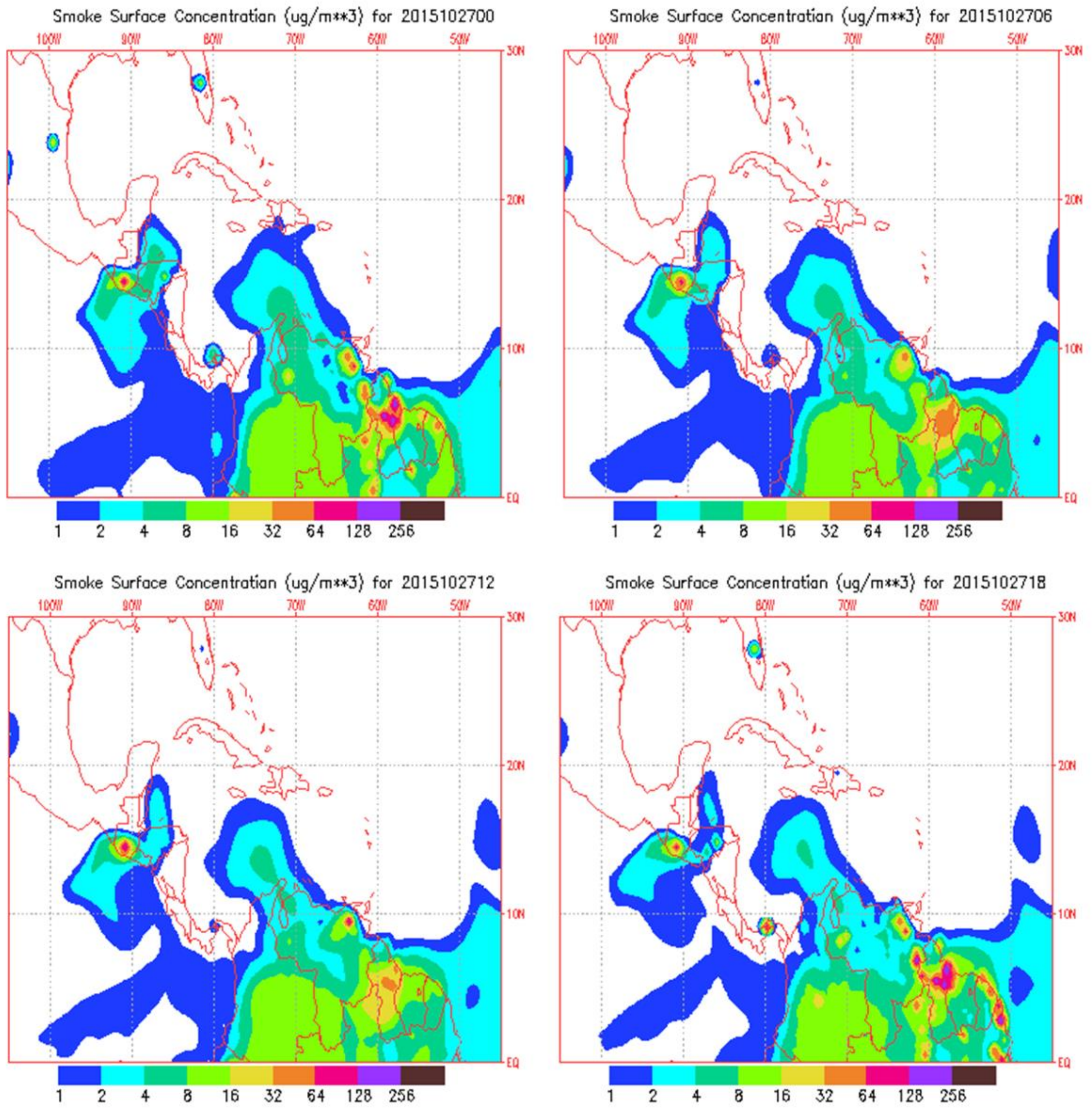

Figure S12. Same as Figure S10 but for 27 October 2015.

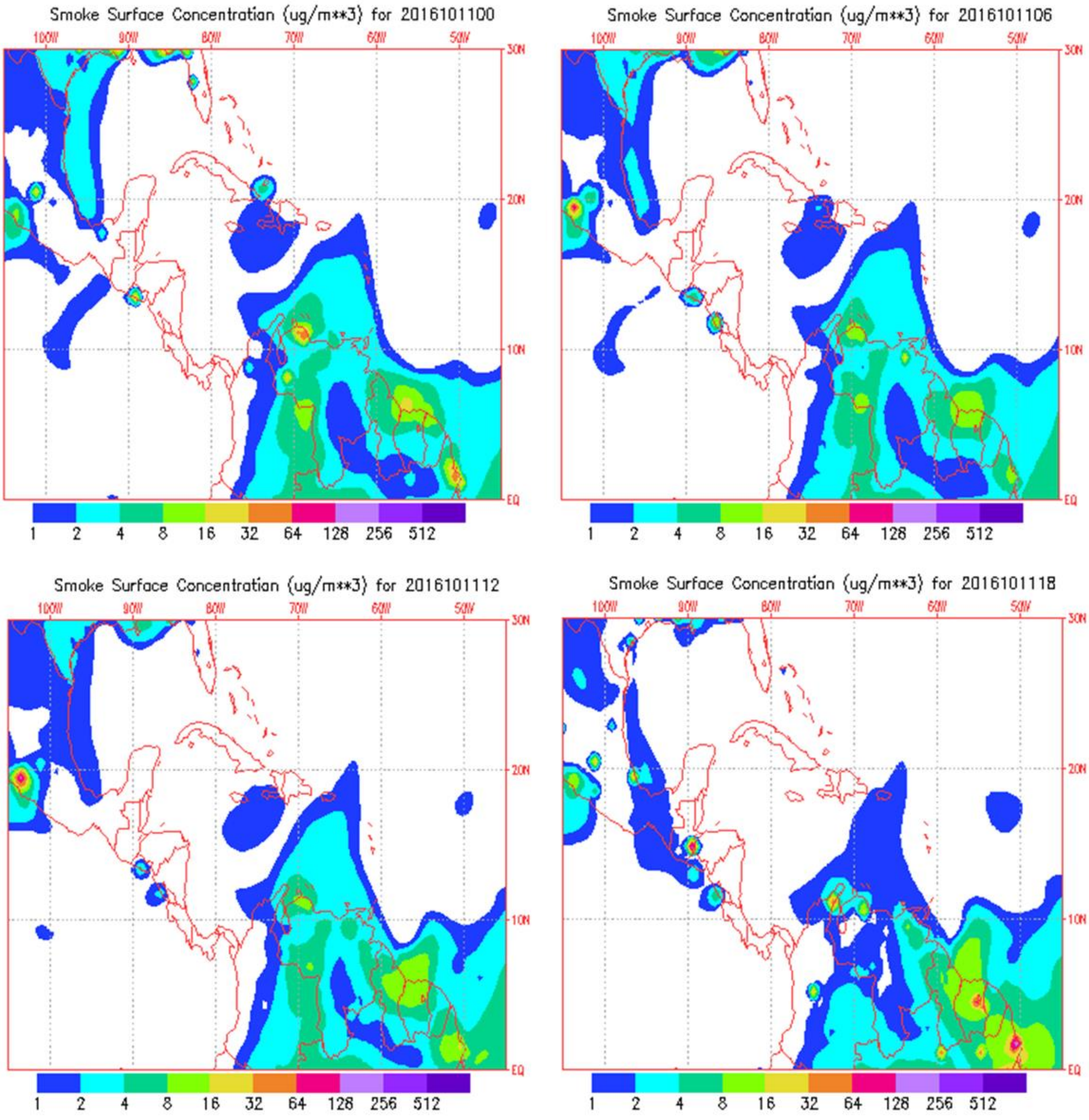

Figure S13. Same as Figure S10 but for 11 October 2016.

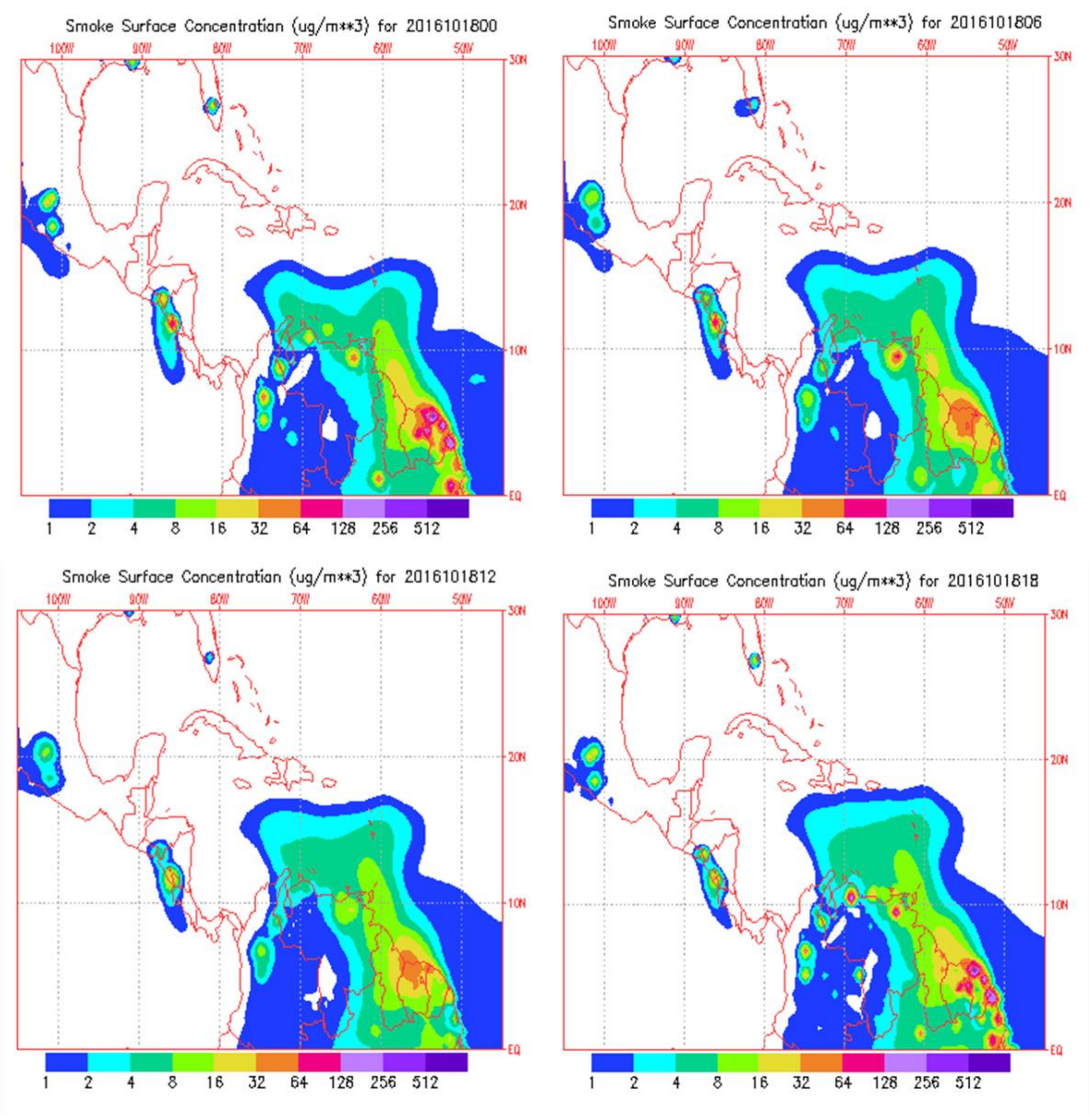

Figure S14. Same as Figure S10 but for 18 October 2016.

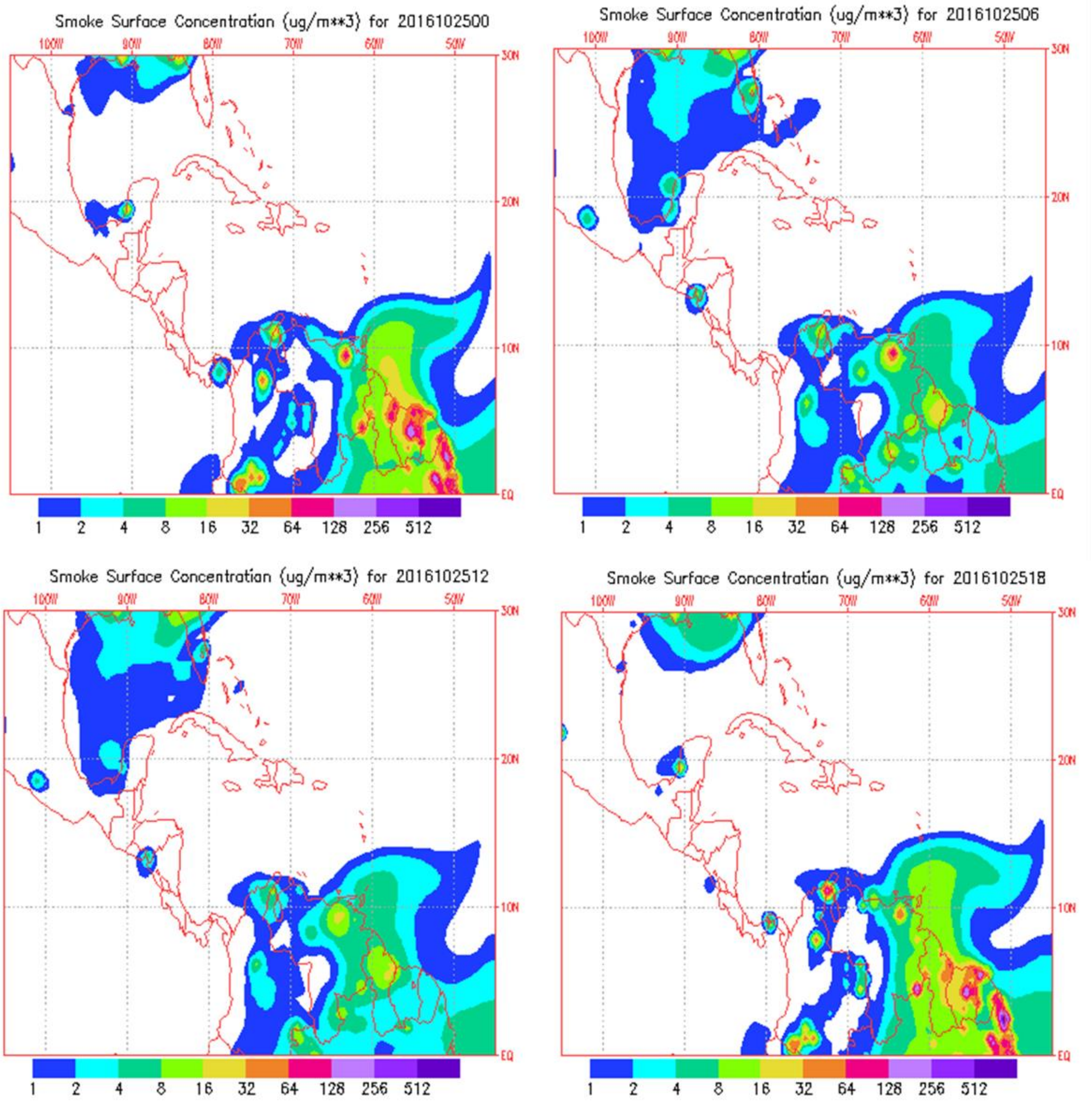

Figure S15. Same as Figure S10 but for 25 October 2016.

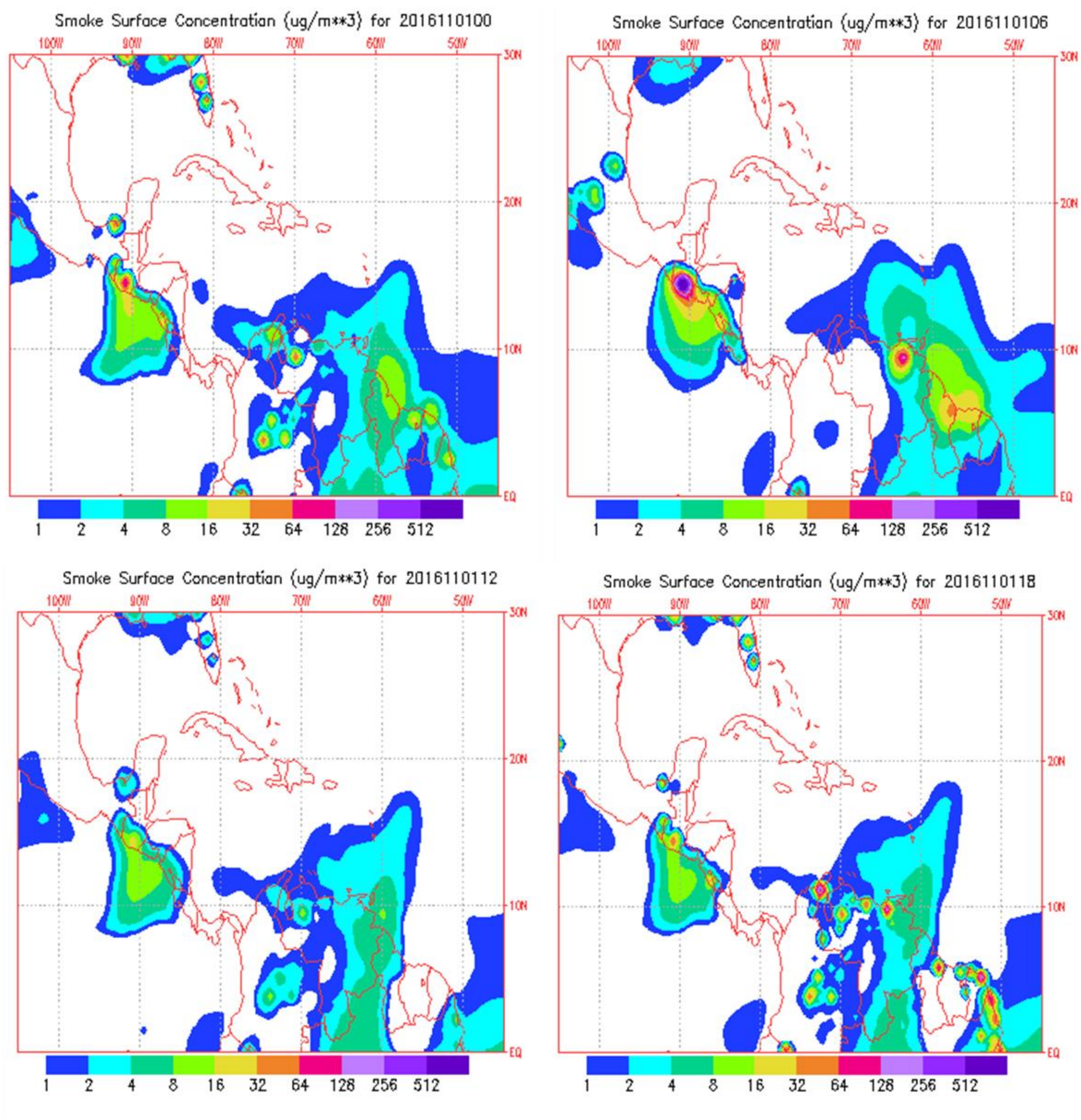

Figure S16. Same as Figure S10 but for 1 November 2016.

References

1. Lynch, P.; Reid, J.S.; Westphal, D.L.; Zhang, J.L.; Hogan, T.F.; Hyer, E.J.; Curtis, C.A.; Hegg, D.A.; Shi, Y.X.; Campbell, J.R., et al. An 11-year global gridded aerosol optical thickness reanalysis (v1.0) for atmospheric and climate sciences. *Geoscientific Model Development* **2016**, *9*, 1489–1522, doi:10.5194/gmd-9-1489-2016.
2. NAAPS. Navy Aerosol Analysis and Prediction System (NAAPS) Global Aerosol Model Availabe online: https://www.nrlmry.navy.mil/aerosol/ (accessed on 1 May 2020).
3. Hogan, T.F.; Liu, M.; Ridout, J.A.; Peng, M.S.; Whitcomb, T.R.; Ruston, B.C.; Reynolds, C.A.; Eckermann, S.D.; Moskaitis, J.R.; Baker, N.L. The navy global environmental model. *Oceanography* **2014**, *27*, 116–125.
4. Cottle, P.; Strawbridge, K.; McKendry, I.; O'Neill, N.; Saha, A. A pervasive and persistent Asian dust event over North America during spring 2010: lidar and sunphotometer observations. *Atmos. Chem. Phys.* **2013**, *13*, 4515–4527, doi:10.5194/acp-13-4515-2013.
5. Lopez, D.H.; Rabbani, M.R.; Crosbie, E.; Raman, A.; Arellano, A.F., Jr.; Sorooshian, A. Frequency and Character of Extreme Aerosol Events in the Southwestern United States: A Case Study Analysis in Arizona. *Atmosphere (Basel)* **2016**, *7*, 1, doi:10.3390/atmos7010001.
6. McKendry, I.G.; Strawbridge, K.B.; O'Neill, N.T.; Macdonald, A.M.; Liu, P.S.K.; Leaitch, W.R.; Anlauf, K.G.; Jaegle, L.; Fairlie, T.D.; Westphal, D.L. Trans-Pacific transport of Saharan dust to western North America: A case study. *J. Geophys. Res. Atmos.* **2007**, *112*, doi:10.1029/2006jd007129.
7. Wells, K.C.; Witek, M.; Flatau, P.; Kreidenwei, S.M.; Westphal, D.L. An analysis of seasonal surface dust aerosol concentrations in the western US (2001-2004): Observations and model predictions. *Atmos. Environ.* **2007**, *41*, 6585–6597, doi:10.1016/j.atmosenv.2007.04.034.
8. Wu, Y.; Han, Z.; Nazmi, C.; Gross, B.; Moshary, F. A trans-Pacific Asian dust episode and its impacts to air quality in the east coast of U.S. *Atmos. Environ.* **2015**, *106*, 358–368, doi:10.1016/j.atmosenv.2015.02.013.
9. Hyer, E.J.; Chew, B.N. Aerosol transport model evaluation of an extreme smoke episode in Southeast Asia. *Atmos. Environ.* **2010**, *44*, 1422–1427, doi:10.1016/j.atmosenv.2010.01.043.
10. Markowicz, K.M.; Lisok, J.; Xian, P. Simulations of the effect of intensive biomass burning in July 2015 on Arctic radiative budget. *Atmos. Environ.* **2017**, *171*, 248–260, doi:10.1016/j.atmosenv.2017.10.015.
11. Dementeva, A.; Zhamsueva, G.; Zayakhanov, A.; Balzhanov, T. Analysis of transport of smoke aerosol in the atmosphere of the Baikal region by data of NAAPS and CALIPSO. In Proceedings of 24th International Symposium on Atmospheric and Ocean Optics: Atmospheric Physics, Tomsk, Russian Federation,December 2018; pp. 1083377, doi: 10.1117/12.2503059.
12. Ge, C.; Wang, J.; Reid, J.S.; Posselt, D.J.; Xian, P.; Hyer, E. Mesoscale modeling of smoke transport from equatorial Southeast Asian Maritime Continent to the Philippines: First comparison of ensemble analysis with in situ observations. *J. Geophys. Res. Atmos.* **2017**, *122*, 5380–5398, doi:10.1002/2016jd026241.
